# Genomic and transcriptomic comparison between *Staphylococcus aureus* strains associated with high and low within herd prevalence of intra-mammary infection

**DOI:** 10.1186/s12866-017-0931-8

**Published:** 2017-01-19

**Authors:** E. Capra, P. Cremonesi, A. Pietrelli, S. Puccio, M. Luini, A. Stella, B. Castiglioni

**Affiliations:** 1 Istituto di Biologia e Biotecnologia Agraria, CNR, via Einstein, 26900 Lodi, Italy; 2 Istituto di Tecnologie Biomediche, CNR, Via Fratelli Cervi 93, 20090 Segrate, Milano Italy; 3 Istituto Zooprofilattico Sperimentale della Lombardia e dell’Emilia, Sezione di Lodi, via Einstein, 26900 Lodi, Italy; 4 Parco Tecnologico Padano, Via Einstein, 26900 Lodi, Italy; 5 Scuola di Dottorato in Medicina Molecolare e Traslazionale, Università di Milano, Segrate, Milan 20009 Italy; 6 Istituto Nazionale di Genetica Molecolare “Romeo ed Enrica Invernizzi”, Via Francesco Sforza 35, 20122 Milan, Italy

**Keywords:** *Staphylococcus aureus*, Mastitis, Virulence, Genome, Transcriptome, Next generation sequencing

## Abstract

**Background:**

*Staphylococcus aureus* (*Staph. aureus*) is one of the major pathogens causing mastitis in dairy ruminants worldwide. The chronic nature of *Staph. aureus* infection enhances the contagiousness risk and diffusion in herds. In order to identify the factors involved in intra-mammary infection (IMI) and diffusion in dairy cows, we investigated the molecular characteristics of two groups of *Staph. aureus* strains belonging to ST8 and ST398, differing in clinical properties, through comparison of whole genome and whole transcriptome sequencing.

**Results:**

The two groups of strains, one originated from high IMI prevalence herds and the other from low IMI prevalence herds, present a peculiar set of genes and polymorphisms related to phenotypic features, such as bacterial invasion of mammary epithelial cells and host adaptation. Transcriptomic analysis supports the high propensity of ST8 strain to chronicity of infection and to a higher potential cytotoxicity.

**Conclusions:**

Our data are consistent with the invasiveness and host adaptation feature for the strains GTB/ST8 associated to high within-herd prevalence of mastitis. Variation in genes coding for surface exposed proteins and those associated to virulence and defence could constitute good targets for further research.

**Electronic supplementary material:**

The online version of this article (doi:10.1186/s12866-017-0931-8) contains supplementary material, which is available to authorized users.

## Background

Mastitis is reported as one of the most important diseases for dairy cattle on the basis of great economical losses caused by affecting animal welfare and milk production costs [1]. *Staphylococcus aureus* is one of the major cause of intra-mammary infection (IMI) in ruminants worldwide, causing mastitis with diverse degrees of severity. In dairy cows, *Staph. aureus* IMI is almost always subclinical, thus leading to an increasing risk of contagion and diffusion in the herds [2]. The molecular pattern of *Staph. aureus* isolates in diverse farm animal forms distinct genetic clusters differing in the presence of pathogenic factors that increase their invasiveness, even in the presence of a stronger inflammatory response [3]. In a recent study [4], subtypes of *Staph. aureus* were associated with high within-herd IMI, compared to other different subtypes that were associated with low within-herd prevalence. This study and previous data [5, 6] confirmed that particular gene patterns, virulence profiles and specific genotypes could be associated with diverse clinical outcomes. More recently, two large European studies [7, 8], demonstrated that the Repetitive-Sequence PCR RS-PCR genotype B (GTB), belonging to the Sequence Type ST8 [9, 10], a high contagious and diffusive *Staph. aureus* involved in bovine IMI, was the most frequently detected in several European countries (Austria, Belgium, France, Germany, Italy, Switzerland). Conversely, the RS-PCR genotype S (GTS), belonging to ST398 [11, 12], was one of the rare genotypes found in bovine milk samples. The ST8 was previously found in both human and dairy cow mastitis [4], suggesting that, after a human-to-cow host jump, a new bovine adaptation took place. At the same time, the ST398 showed a host transition from human to animal reservoir, becoming the most widely disseminated clonal complex in bovine species and in the milk samples collected in herds with low prevalence of IMI [13–15].

The existence of subtypes of *Staph. aureus* differing in pathogenic properties emphasizes the need to well define strain characteristics, in order to monitor bacteria dissemination and find potential relevant targets related to their contagiousness. In recent years the advent of next generation sequencing (NGS) technologies has improved the estimate of the correlation of virulence phenotype to genome structure, providing a more detailed picture of gene patterns involved into staphylococcal pathogenesis. High-throughput whole-genome sequencing of *Staph. aureus* was prevalently used to monitor outbreaks in hospitals [16, 17], to evaluate strains transitioning from carrier to invasive *status* [18] and to understand aspects of pathogen biology in detailed epidemiological studies in human [19–21]. In livestock community, the molecular basis of virulence in *Staph. aureus* mastitis was investigated by using an integrated approach that includes NGS, microarray and proteomic data [22] providing the first high-resolution comparison between gene content and gene expression in two *Staph. aureus* strains. More recently, Peton and co-workers [23] described a fine-tuned characterization of *Staph. aureus* Newbond 305, a strain belonging to ST115 and associated to bovine mastitis, by genomic and proteomic comparison with the reference strain RF122. Gene expression analysis by microarray techniques has provided, also, information about global transcript changes [24, 25] or molecular basis of virulence [26] in *Staph. aureus*. Moreover, RNA-seq was recently used to study (i) the gene expression in different *Staph. aureus* strains [27, 28], (ii) the role of anti-sense transcription [29] and (iii) the identification of small non-coding RNAs [30].

To gain further insight into *Staph. aureus* features, the aim of this work was the characterization of two groups of *Staph. aureus* strains differing in their clinical outcome. Each strain was comprehensively studied by comparative genomic and transcriptomic analysis in order to identify staphylococcal factors that can be associated with strain virulence and bacterial diffusion in the herd.

## Methods

### Bacterial strains

Six bacterial strains, originally isolated from subclinical cases of bovine IMI in six different Holstein herds (A-F, Table 1) located in Lombardy region in the northern of Italy, were used in this study. The average size of the herds was 106 milking cows (range 38 to 285 cows). Milk samples were collected aseptically. Samples were kept at 4 °C and bacteriological assays were performed within 48 h. Isolates were classified into two groups: *Staph. aureus* belonging to low within-herd mastitis prevalence (herds A, B, C) or high within-herd mastitis prevalence (herds D, E, F). As described in Table 1, and reported by Cremonesi and colleagues [4], *Staph. aureus* isolates had been previously characterized by RS-PCR, Multi Locus Sequence Type (MLST) [31], for presence of *mec*A gene and for different virulence genes. The strains collected in three different herds with low mastitis prevalence (between 2 and 4%) were identified by RS-PCR as genotype S (GTS) and by MLST as ST398 (hereinafter referred as GTS/ST398). Two out of three were positive for *mec*A gene. The strains isolated from three herds with high IMI prevalence (between 49 and 62%) were identified by RS-PCR as genotype B (GTB) and ST8 (hereinafter referred as GTB/ST8) and none of them harboured the *mec*A gene coding for methicillin resistance.

**Table 1:** Characteristics of the bacterial strains used in this study

	Herd	IMI prevalence	MLST	RS-PCR	mecA	Virulence profile*
GTS/ST398						
Strain 1	A	4%	ST398	GTS	+	*luk*E, *cna*, *fmtb*, *scn*, *chp*, *luk*M
Strain 2	B	2%	ST398	GTS	-	*clf*A, *luk*E, *cna*, *fmtb*, *scn*, *chp*, *luk*M
Strain 3	C	2%	ST398	GTS	+	*clf*A, *luk*E, *cna*, *fmtb*, *luk*M
GTB/ST8						
Strain 1	D	49%	ST8	GTB	-	*clf*A, *luk*E, *cna*, *sea*, *sed*, *sej, fmtb*, *scn*, *chp*
Strain 2	E	54%	ST8	GTB	-	*clf*A, *luk*E, *cna*, *sea*, *sed*, *sej, fmtb*, *scn*, *chp*
Strain 3	F	62%	ST8	GTB	-	*clf*A, *luk*E, *cna*, *sed*, *sej*, *seg*, *sei*, *fmtb*, *chp*

Herd Isolation (six different herds named A-F), IMI prevalence, sequence type characterization by MLST, RS-PCR analysis (genotype S, GTS; genotype B, GTB), *mec*A detection and virulence genes analysis (*lukE, leucotoxin E gene; *cna*, collagen adhesin-encoding gene; *fmt*b, gene encoding for cell wall-associated protein; *scn*, staphylococcal complement inhibitor gene; *chp*, chemotaxis inhibitory protein gene; *luk*M, leukotoxin M gene; *clf*A, clumping factor A gene; *sea*, enterotoxin A; *sed*, enterotoxin D; *sej*, enterotoxin J; *seg*, enterotoxin G, *sei*, enterotoxin I)

### Growth conditions

The strains were isolated and grown on Blood Agar plates and a single colony of the third passage in culture was transferred into 5 ml of Brain Heart Infusion medium (BHI). Bacteria were grown overnight at 37 °C. Cultures were subsequently diluted 1/100 into 40 ml of BHI and grown at 37 °C. Optical density at 600 nm (OD_600_) was performed hourly until mid-exponential phase (OD_600_ = 0.4) was achieved. At the appropriate OD_600_, bacteria were pelleted by centrifugation at 10 000 g for 2 min; after surnatant removing, the pellet was resuspended in 500 μl of saline solution (NaCl 0.9%) and centrifuged at 10 000 g for 2 min. The pellet was immediately used for RNA extraction and stored at −20 °C for DNA extraction.

### Bacterial DNA and RNA extraction

Genomic bacterial DNA was extracted using the protocol previously described [32], starting from step 2. Total RNA was isolated using the NucleoSpin® mRNA kit (Macherey-Nagel, Germany), according to the manufacturer protocol, in combination with TRIzol® lysis. DNAs and RNAs were quantified using a NanoDrop ND-1000 spectrophotometer (NanoDrop Technologies, Wilmington, DE, USA) and RNAs quality was checked using the Agilent Bioanalyser 2100 (Agilent, Santa Clara, CA). Only RNA samples with RNA Integrity Number (RIN) values higher than 6.5 were used for the analysis. The isolated DNAs and RNAs were stored at −20 and −80 °C until use, respectively.

### Library preparation and Miseq sequencing

#### DNA

Libraries were constructed using TruSeq PCR free Kit (Illumina, San Diego, CA, USA) following the manufacturer’s instructions, sequenced in one 2 × 300-cycles Miseq run (Illumina, San Diego, CA, USA).

#### RNA

RNA was processed as previously described [27] with some variations. Briefly, bacterial rRNA was depleted with RiboZero rRNA removal kit for gram-positive organisms (Epicentre Illumina, Madison, WI, USA). RNA quality was assessed for each passage by the Agilent Bioanalyser 2100 (Agilent, Santa Clara, CA). Libraries were prepared using TruSeq® RNA Sample Preparation v2 Kit (Illumina). Samples were sequenced on a Miseq Instrument (Illumina) in a 1 × 50-cycles run.

### Bioinformatics analysis

*Staph. aureus* NCTC 8325 core gene evaluation and De-Novo Assembly of GTB/ST8 and GTS/ST398 strains. The quality of the raw sequencing reads was assessed by using FastQC software (http://www.bioinformatics.babraham.ac.uk/projects/fastqc/). Adapter removal and quality trimming has been performed using Trimmomatic [33], with default parameters and nucleotide PHRED quality > 30.

High-quality reads were mapped against the reference genome of NCTC 8325. BWA has been used as mapping software to detect common genes between GTS/ST398, GTB/ST8 and NCTC 8325. GTS/ST398 and GTB/ST8 reads were cross-mapped against NCTC 8325 genome. We applied quality filters by excluding those reads with more than four mismatches or those with mapping quality score (MAPQ) less than 15 in the resulting BAM files. To select genes present in the three genomes, we applied filters on coverage and depth. Briefly, only those genes that present 100% length coverage and a minimum 10X mean depth in the two mapping strains have been selected (Additional file 1). 2478 genes were selected and used in RNASeq analysis for quantification and differential expression. To perform the assembly the short-read assembly tool SPAdes 3.1.1 [34] was used. To obtain a reference assembly (ra) for each group of strains (genotype GTS/ST398 and GTB/ST8), the single assemblies (three for each group), were merged with CISA [35]. GTB/ST8 reference assembly (GTB/ST8ra) and GTS/ST398 reference assembly (GTS/ST398ra) were annotated with RAST [36]. To overcome false protein duplication and misassembly issues, we performed a reciprocal BLASTp within GTB/ST8ra set and GTS/ST398ra set separately. Whether a protein presented a perfect match (100% sequence identity and 100% length identity) with another one, only one of them was selected.

The protein sequences comparison between the strains (GTS/ST398, GTB/ST8) and the definition of the “core” (set of genes shared between GTB/ST8ra and GTS/ST398ra) and “accessory” (set of unique genes for both genotypes) genomes was performed by using In Paranoid 4.1 software [37], a BLAST-based algorithm to compute protein homology analysis between two or more species. For GTB/ST8 and GTS/ST398 analysis, a cut-off of 0.9 for sequences overlap and the default values for the other options were used. The functional enrichment analysis was performed using the Fisher’s test on the functional categories after Bonferroni multiple testing correction using R software version 3.0.3.

#### Genomic comparison with other *Staph. aureus* reference strains

For comparative analysis, reference genome sequences of 22 strains available in NCBI were used (Additional file 2). The genome similarities based on phylogenetic distances were analyzed using the Gegenees software [38]. A fragmented alignment in TBLASTX mode was performed with settings 500/500 and dendrogram was produced in SplitsTree 4 [39]. Visualization of genome comparisons was performed using BLAST Ring Image Generator [40].

#### Transcriptomic analysis

Transcriptome reads were mapped against the reference sequence of *Staph. aureus* NCTC 8325 genome with BWA aligner [41]. To generate a high-quality mapping for each sample, we applied quality filters by excluding those reads with more than four mismatches or those with mapping quality score (MAPQ) less than 15. Read counts for gene relative abundance, differential expression analysis and statistical analysis were calculated as previously described [42, 43].

Differential expression analysis was performed on the gene set belonging to core genome of *Staph. aureus* NCTC8325 and the calculation of differential expression genes was performed with DESeq [44]. Differential expressed genes were selected with specific filters: 1) *p*-value less than 0.01 after Bonferroni correction (padj) 2) log2FC >1.5 or < -1.5. Functional categories annotation for each gene was extracted from COG database [45] and the Fisher’s test was used for enrichment analysis after Bonferroni correction. All the statistical analyses were performed using R version 3.0.3. DNA-Seq and transcriptomic data were visualized using the Integrated Genomics Viewer IGV [46]. Transcriptomic data are available in Sequence Reads Archive (SRA) accession number SRX965931.

#### Real Time PCR *qRT-PCR*

Primers used for real-time PCR were designed using Primer Express software V2.0 (Applied Biosystems, Foster City, CA) and are listed in (Additional file 3). Pyrroline-5-carboxylate reductase (*proC*) was used as a reference gene [47]. Each sample was treated with DNAse and cDNAs were synthesized using GoScript™ Reverse Transcription System (Promega, Madison, WI) with random primers following manufacturer instruction. Real-time PCR was performed with 7900HT Fast Real-Time PCR System (Applied Biosystems, Carlsbad, California, USA) using *Power* SYBR® Green PCR Master Mix (Applied Biosystems) according to manufacturer protocols. Data were analyzed with Sequence Detection Systems SDS Software (version 2.3).

#### Pathway analysis

Protein sequences from unique GTB/ST8 and GTS/ST398 and differential expressed genes (DEGs) were used as queries in KOALA (KEGG Orthology And Links Annotation) tool for pathways reconstruction [48].

#### *fnbB* partial re-sequencing

Primers fnbB-F1 (5′-TTCTGCATGACCTTCTGCAC-3′) and fnbB-R1 (5′-AGCAAGCGAAACACAAACAA-3′) were used to amplify a portion from 1222 up to 2656 bp of *fnbB* genes (NCBI accession number: CP000253, region: 2577879......2580632) in all the six strains. PCR was performed in a final volume of 25 μl, containing ~60 ng of DNA, 0.8 μM of each primer, 12.5 μl of GoTaq® Long PCR Master Mix (Promega, Italy), with the following cycling parameters: 95 °C for 2 min, 30 cycles of 94 °C for 30 s, 56 °C for 30 s and 72 °C for 2 min; and then 72 °C for 10 min. PCR products were loaded in 1.5% agarose gel. PCR products were purified with Wizard Clean-up (Promega, Italy), following manufacturer′s instructions. Purified PCR products were sequenced bi-directionally (GATC Biotech, Konstanz, Germany) with primer fnbB-F1 and fnbB-R1. *fnbB* partial sequences determined in this study have been submitted to GenBank with accession numbers KY024702 and KY024703 for GTB/ST8 and GTS/ST398, respectively.

## Results

In this study six strains of *Staph. aureus* previously described [4] as strictly associated with high and low within-herd IMI prevalence, respectively, were analysed in order to discover, thanks to comparative genomics and transcriptomics, potential pathogenic factors associated with the different clinical outcome found in the herds.

### Sequencing of GTB/ST8 and GTS/ST398 genotypes

#### Genome assembly and Comparative Genomics

Genomic diversity between the *Staph. aureus* GTB/ST8 and GTS/ST398 genotypes was assessed analyzing three DNA samples for each genotype that were deeply sequenced with an average production of 2,908,485 (max 4,630,318 and min 1,399,737) reads per sample. The sequencing reads from every sample were assembled obtaining an average of 95 number of large contigs (>500 nt) from all the samples (mean GTB/ST8: 50; mean GTS/ST398: 139) (Additional file 4).

**Fig. 1:**
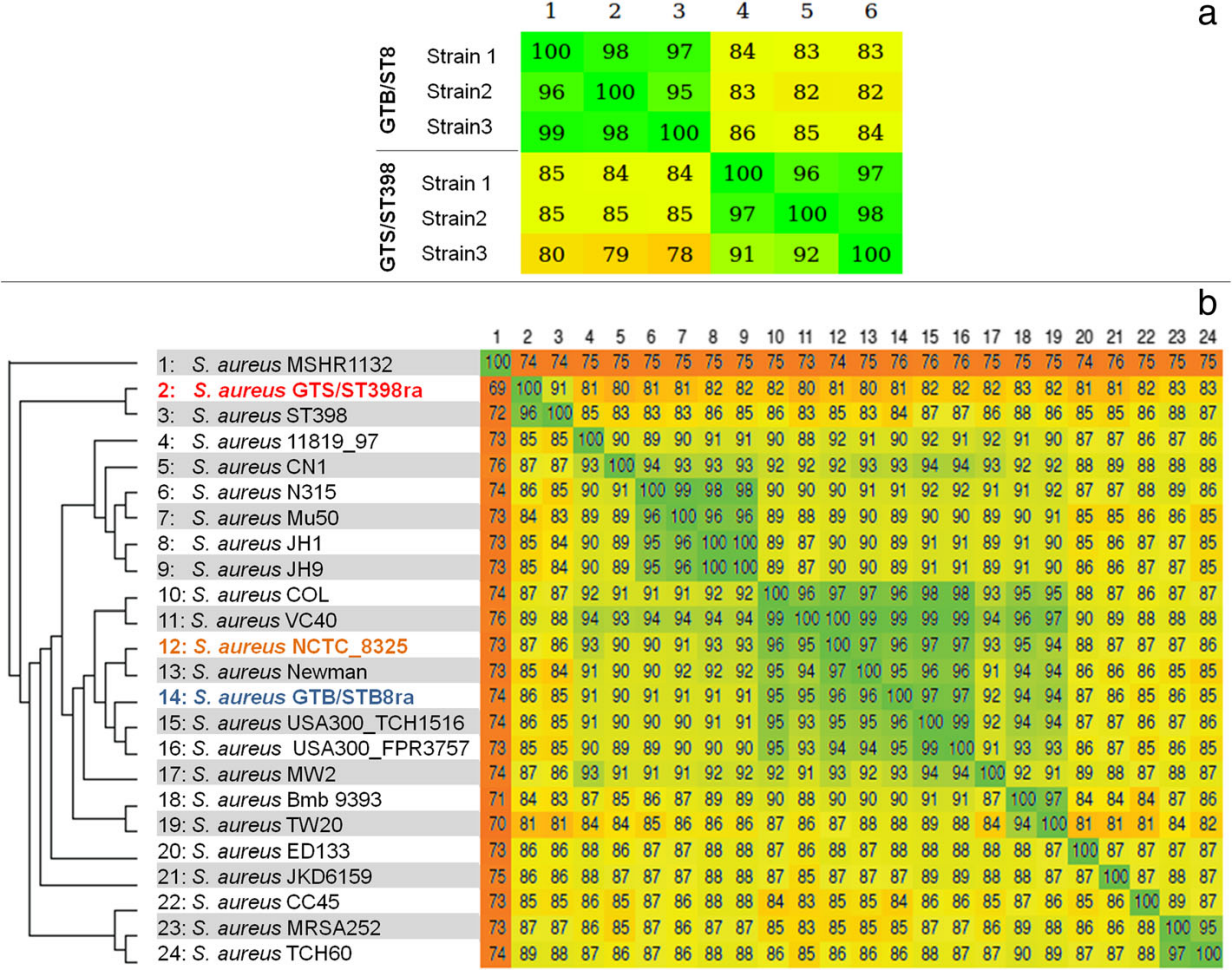
**a** Percentage of similarity between the three GTB/ST8 and the three GTS/ST398strains. **b** Phylogenesis distances and percentage of similarity between the two reference assemblies GTB/ST8ra (blue) GTS/ST398ra (red) and other *Staph. aureus* strains. In brown the NCTC_8325 strain used as reference for RNASeq data analysis

Three GTB/ST8 and three GTS/ST398 assemblies showed a high level of within group similarity, ranging from 91 to 99% and 95 to 98% respectively, whereas only a partial similarity (from 78 to 86%) was observed between the two groups (Fig. 1a).

To create a single reference genome representing each group, all the single genotype-specific assemblies were merged together producing two reference assemblies, one for GTB/ST8 and one for GTS/ST398 with 19 (3.366.835 nt) and 291 (3.284.103 nt) large contigs, respectively. These reference assembly genomes, named GTB/ST8ra and GTS/ST398ra, respectively, were used for the genomic analysis in comparison with the genomes of other 22 *Staph. aureus* reference strains, fully sequenced and available in GenBank (Fig. 1b). The two genotypes GTS/ST398ra and GTB/ST8ra here analysed clustered better with the *Staph. aureus* ST398 prototype and *Staph. aureus* TCH1516, respectively, the latter being a human ST8 reference strain.

#### GTB/ST8ra and GTS/ST398ra genome comparison

**Fig. 2:**
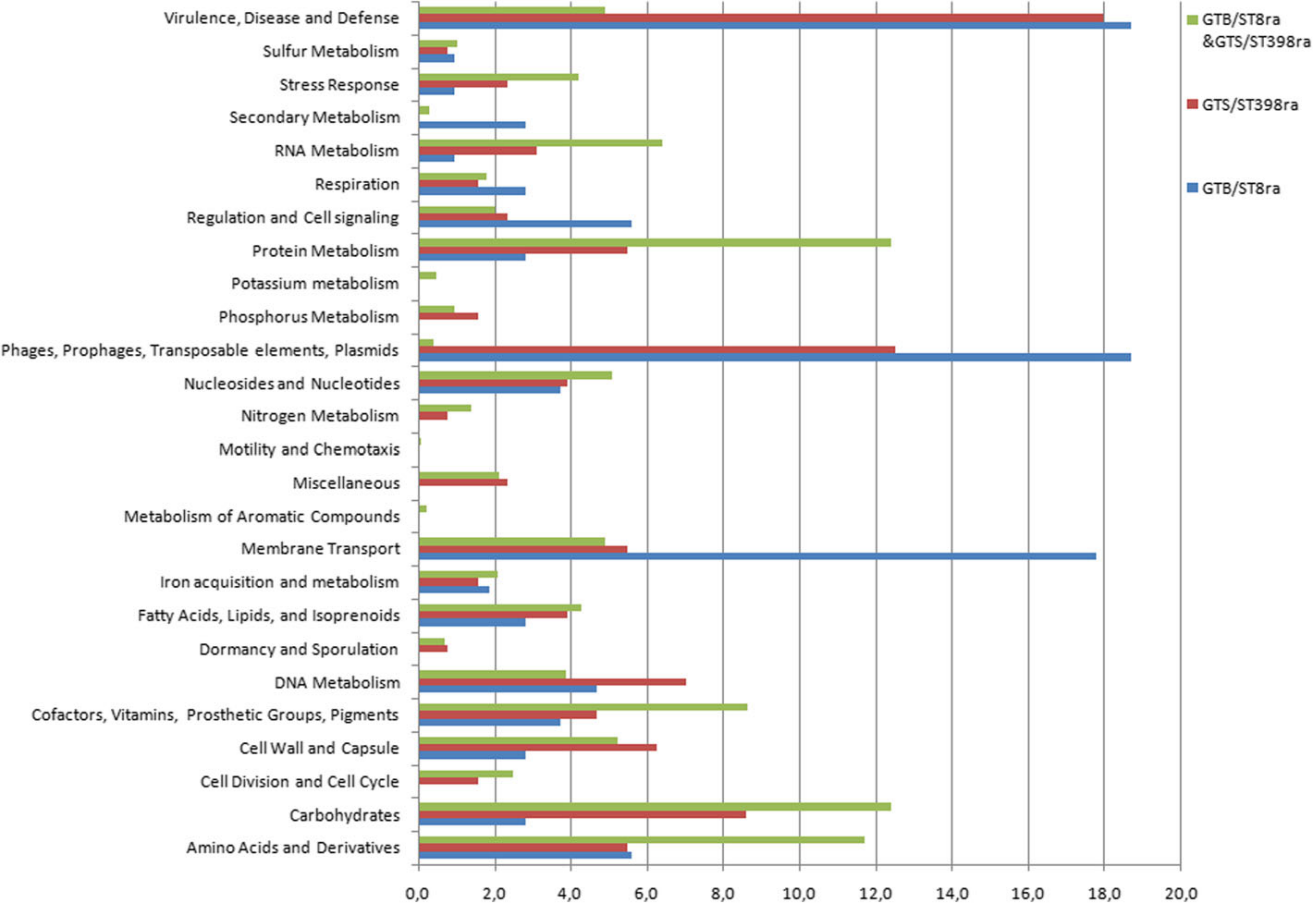
Gene ontology (GO) categories comparison between annotated genes belonging to the core genome (shared GTB/ST8ra&GTS/ST398ra genes, in green) and unique gene for GTB/ST8ra (blue) and GTS/ST398ra (brown). X axis indicates the percentage value of genes belonging to each categories reported

Two thousand six hundred sixty-seven and 2712 Coding DNA Sequences (CDS) were annotated from the prediction tool for GTB/ST8ra and GTS/ST398ra, respectively. Predicted proteins were functionally categorized using the COGs database. As expected, about 50% were annotated with a functional role (48.74% GTB/ST8ra and 48.48% GTS/ST398ra, respectively). Protein homology analysis revealed that the majority of CDSs (*n* = 2247) was shared between the two groups of strains, since up to 84.25% and 82.85% of the CDSs belonged to the core genome of GTB/ST8ra and GTS/ST398ra, respectively (Additional file 5). The COGs distributions were similar in the two genomes: both GT8/ST8ra and GTS/ST398ra presented a set of unique genes belonging to “Phages, Prophages, Transposable elements, Plasmids” and “Virulence, Disease and Defence” categories that were significantly enriched (*p*-value < 0.01) compared to the core genome. In addition, GTB/ST8ra was significantly enriched also in “Membrane Transport” genes (Fig. 2, Additional file 6). For both groups of strains, unique genes associated to virulence were prevalently attributed to “Adhesion function” and “Resistance to antibiotics and toxic compounds and toxin production” (Table 2).

**Table 2:** Genes associated to virulence resulting different between the two reference genotypes (GTB/ST8ra and GTS/ST398ra)

Sequence annotation (Features)	Subcategory	Function	GTB/ST8ra	GTS/ST398ra
fig|6666666.84847.peg.				
1289	1	Extracellular adherence protein of broad specificity *Eap*/*Map*	+	−
1290	1	Extracellular adherence protein of broad specificity *Eap*/*Map*	+	−
150	2	Fosfomycin resistance protein *FosB*	+	−
1807	1	Virulence-associated cell-wall-anchored protein SasG (LPXTG motif)	+	−
1812	1	Fibronectin binding protein *Fnb*B	+	−
2863	1	Adhesin of unknown specificity *Sdr*C	+	−
2986	2	Arsenate reductase (EC 1.20.4.1)	+	−
3003	1	Predicted cell-wall-anchored protein *Sas*C (LPXTG motif)	+	−
311	1	Virulence-associated cell-wall-anchored protein *Sas*G (LPXTG motif)	+	−
312	1	Virulence-associated cell-wall-anchored protein *Sas*G (LPXTG motif)	+	−
3121	3	Leukotoxin *Luk*D	+	−
3122	3	Leukotoxin *Luk*E	+	−
316	1	Fibronectin binding protein *Fnb*B	+	−
564	1	Virulence-associated cell-wall-anchored protein *Sas*G (LPXTG motif)	+	−
582	2	Arsenate reductase (EC 1.20.4.1)	+	−
583	2	Arsenic efflux pump protein	+	−
584	2	Arsenical pump-driving ATPase (EC 3.6.3.16)	+	−
586	2	Arsenical resistance operon repressor	+	−
618	1	Protein A, von Willebrand factor binding protein *Spa*	+	−
930	1	Cadmium resistance protein	+	−
fig|6666666.84857.peg.				
100	2	Predicted cell-wall-anchored protein *Sas*A (LPXTG motif)	−	+
1028	1	Tetracycline resistance protein *TetM*	−	+
1345	2	Adhesin of unknown specificity *SdrE*	−	+
1462	1	Two-component sensor histidine kinase *BceS*	−	+
1498	4	Protein A, von Willebrand factor binding protein *Spa*	−	+
1574	1	TetR family regulatory protein of MDR cluster	−	+
1856	2	Collagen binding protein *Cna*	−	+
2046	1	Fibronectin binding protein *Fnb*B	−	+
2047	1	Fibronectin binding protein *Fnb*B	−	+
2308	1	Predicted cell-wall-anchored protein *Sas*C (LPXTG motif)	−	+
2309	1	Predicted cell-wall-anchored protein *Sas*C (LPXTG motif)	−	+
267	1	Virulence-associated cell-wall-anchored protein SasG (LPXTG motif)	−	+
2840	1	Copper-translocating P-type ATPase (EC 3.6.3.4)	−	+
2903	2	Collagen binding protein *Cna*	−	+
317	1	Spectinomycin 9-O-adenylyltransferase	−	+
334	2	Clumping factor ClfB, fibrinogen binding protein	−	+
344	1	Adhesin of unknown specificity *Sdr* *E*	−	+
352	1	Predicted cell-wall-anchored protein *Sas*A (LPXTG motif)	−	+
510	1	Adhesin of unknown specificity *SdrE*	−	+
512	1	Adhesin of unknown specificity *SdrC*	−	+
561	1	Spectinomycin 9-O-adenylyltransferase	−	+
609	2	Extracellular adherence protein of broad specificity *Eap*/*Map*	−	+
63	1	Collagen binding protein Cna	−	+

(+) presence, (−) absence. The sequence annotation (Features) subcategory were: 1) Adhesion, 2) Resistance to antibiotics and toxic compounds, 3) Toxins and superantigens and 4) Bacteriocins, ribosomally synthesized antibacterial peptides. For each entry the Function was reported

### GTB/ST8 and GTS/ST398 transcriptomic comparison

#### Transcriptomic analysis and *qRT-PCR validation*

RNA-Seq data covering the *Staph. aureus* genome were used to quantitatively compare gene expression levels between the two groups of strains grown in the exponential phase. Transcriptome reads were aligned against *Staph. aureus* NCTC8325 reference genome with an high read-mapping rate and high coverage for both genotypes (average: 93.25% ± 1.39%) (Additional file 7). Among the 2479 genes that were in common between the three genomes (GTS/ST398ra, GTB/ST8ra and NCTC8325), 237 differential expressed genes (DEGs) were found between the two groups with a distribution of 56.1% of the DEGs up-regulated in GTB/ST8 strains and 43.9% in GTS/ST398 strains (Additional file 8).

Four of these differentially expressed genes were validated by RT-PCR: SAOUHSC_00773, the *Lys* *M* domain-containing protein; SAOUHSC_01181 an hypothetical proteins; SAOUHSC_01314, DNA-binding response regulator; SAOUHSC_01450the basic amino acid/polyamine antiporter, APA family protein. For each test, qPCR results confirmed RNA-Seq data: SAOUHSC_01314 not expressed in one of the two genotypes resulted in undetermined CT value, whereas the three differentially expressed genes SAOUHSC_00773, SAOUHSC_01181, SAOUHSC_01450 showed fold changes of 0.0538, 9.0972 and 0.1297 in qPCR respectively, comparable with the fold changes of 0.0694, 91.4000 and 0.0265 obtained from RNA-Seq (Additional file 9a, b).

#### Functional analysis of transcriptomic data

**Fig. 3:**
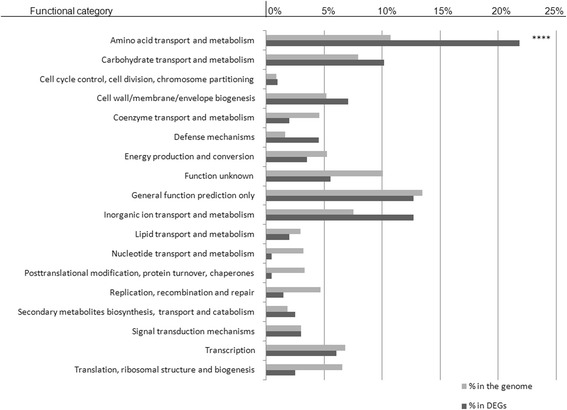
Gene ontology (GO) categories comparison between significant DEGs (in grey) and genes expressed in the *Staph. aureus* NCTC_8325 strain (in black). ****P ≤ 1e-4

The comparison between the number of DEGs and the total number of genes present in the NCTC8325 reference genome, revealed that genes belonging to “Amino acid transport and metabolism” category varied significantly between the two genotypes (corrected *p*-value 3.86E-05). Other two categories, “Defence mechanism” and “Inorganic ion transport and metabolism” indicated a trend toward enrichment in DEGs list (Fig. 3).

Within the 42 annotated genes related to “Aminoacid transport and metabolism” pathway, only 9 were up-regulated in GTB/ST8 (Table 3). Concerning “Defence mechanism” we found 34 annotated genes, within 14 and 20 up-regulated in GTB/ST8 and GTS/ST398, respectively. Finally, among the 24 DEGs belonging to “Inorganic ion transport and metabolism”, 4 and 20 were up-regulated in GTB/ST8 and GTS/ST398, respectively (Table 3).

**Table 3:** DEGs between GTB/ST8 and GTS/ST398 strains

LocusTag SAOUHSC	Function	Protein Description	padj	log2 FC	GTB/ST8	GTS/ST398
_01451	1	catalyzes the formation of 2-oxobutanoate from L-threonine; catabolic	1.28E-56	−5.35	↓	↑
_01450	1	Amino acid transporters	3.35E-52	−5.25	↓	↑
_01452	1	alanine dehydrogenase	1.34E-50	−5.16	↓	↑
_01448	1	Permeases of the major facilitator superfamily	1.82E-41	−5.16	↓	↑
_02969	1	catalyzes the degradation of arginine to citruline and ammonia	3.51E-22	−4.34	↓	↑
_02968	1	catalyzes the formation of ornithine and carbamylphosphate from citrulline in the arginine catabolic pathway	2.42E-15	−3.62	↓	↑
_00108	1	Zinc peptidase	8.34E-11	−6.03	↓	↑
_02967	1	arginine/ornithine antiporter, putative	2.00E-09	−2.65	↓	↑
_01803	1	Gamma-aminobutyrate permease and related permeases	2.86E-08	2.41	↑	↓
_02767	1	peptide ABC transporter, peptide-binding protein, putative	3.75E-08	−2.17	↓	↑
_02435	1	Permeases of the major facilitator superfamily	2.63E-07	−2.42	↓	↑
_01307	1	Threonine aldolase	1.95E-06	−3.19	↓	↑
_02766	1	peptide ABC transporter, permease protein, putative	3.06E-05	−1.95	↓	↑
_02763	1	peptide ABC transporter, ATP-binding protein, putative	5.33E-05	−1.97	↓	↑
_01825	1	Cysteine sulfinate desulfinase/cysteine desulfurase and related enzymes	5.72E-05	1.95	↑	↓
_01320	1	catalyzes the formation of L-aspartate 4-semialdehyde from L-homoserine	5.97E-05	−1.71	↓	↑
_00076	1	ornithine cyclodeaminase, putative	6.44E-05	−2.70	↓	↑
_01833	1	catalyzes the formation of 3-phosphonooxypyruvate from 3-phospho-D-glycerate in serine biosynthesis;	7.00E-05	−1.77	↓	↑
_02740	1	drug transporter, putative	0.000142	1.65	↑	↓
_02697	1	amino acid ABC transporter, ATP-binding protein, putative	0.000231	1.57	↑	↓
_02825	1	Lactoylglutathione lyase and related lyases	0.000256	−2.77	↓	↑
_00733	1	catalyzes the formation of L-histidinol phosphate in histidine biosynthesis	0.000257	−1.78	↓	↑
_01321	1	catalyzes the formation of L-threonine from O-phospho-L-homoserine	0.000283	−1.60	↓	↑
_00075	1	siderophore biosynthesis protein SbnA	0.00032	−2.62	↓	↑
_02559	1	ureases	0.00039	−2.37	↓	↑
_01991	1	ABC transporter, permease protein, putative	0.00047	1.83	↑	↓
_01991	1	ABC transporter, permease protein, putative	0.00047	1.83	↑	↓
_02765	1	nickel ABC transporter, permease protein, putative	0.000471	−1.75	↓	↑
_02433	1	Predicted amino acid racemase	0.000512	−1.60	↓	↑
_02764	1	peptide ABC transporter, ATP-binding protein, putative	0.000687	−1.70	↓	↑
_02561	1	ureases	0.000724	−1.65	↓	↑
_00421	1	Cysteine synthase	0.000785	−1.60	↓	↑
_01319	1	catalyzes the formation of 4-phospho-L-aspartate from L-aspartate and ATP; lysine and threonine sensitive	0.000879	−2.01	↓	↑
_00740	1	Permeases of the drug/metabolite transporter (DMT) superfamily	0.001181	1.71	↑	↓
_02839	1	L-serine dehydratase, iron-sulfur-dependent, alpha subunit	0.001587	−1.51	↓	↑
_01395	1	aspartate-semialdehyde dehydrogenase	0.001826	−1.64	↓	↑
_00703	1	quinolone resistance norA protein, putative	0.002597	1.67	↑	↓
_02558	1	UreA, with UreB and UreC	0.005062	−2.29	↓	↑
_01990	1	amino acid ABC transporter, ATP-binding protein, putative	0.005951	1.50	↑	↓
_02932	1	catalyzes the oxidation of choline to betaine aldehyde and betain aldehyde to glycine betaine	0.006268	−1.53	↓	↑
_01394	1	catalyzes the formation of 4-phospho-L-aspartate from L-aspartate and ATP, in Bacillus, lysine sensitive; regulated by response to starvation,	0.006704	−1.68	↓	↑
_00170	1	peptide/nickel transport system substrate-binding protein	0.008873	−1.55	↓	↑
_02972	2	immunodominant antigen B IsaB	6.70E-24	5.79	↑	↓
_01079	2	neurofilament protein	7.69E-23	−4.51	↓	↑
_02127	2	staphopain thiol proteinase	4.50E-18	−3.61	↓	↑
_01933	2	type I restriction-modification system, M subunit	5.20E-18	−5.10	↓	↑
_02708	2	gamma-hemolysin h-gamma-ii subunit, putative	4.61E-17	−3.60	↓	↑
_01115	2	staphylococcal complement inhibitor SCIN	3.05E-15	5.06	↑	↓
_01135	2	anti protein (phenol soluble modulin)	2.18E-13	−3.07	↓	↑
_01964	2	signal transduction protein TRAP	3.38E-13	3.35	↑	↓
_00668	2	ABC transporter permease, putative	3.60E-13	3.99	↑	↓
_02420	2	multidrug resistance protein SepA	2.58E-11	3.99	↑	↓
_02963	2	clumping factor B, putative	2.92E-11	2.75	↑	↓
_02419	2	methicillin resistance protein FmtB	2.69E-10	5.00	↑	↓
_00256	2	staphyloxanthin biosynthesis protein, secretory antigen precursor SsaA	1.48E-09	−2.69	↓	↑
_02802	2	fibronectin binding protein B, putative	3.09E-09	2.32	↑	↓
_02709	2	leukocidin s subunit precursor, putative	1.30E-08	−2.45	↓	↑
_02710	2	leukocidin f subunit precursor	1.50E-08	−2.43	↓	↑
_01084	2	Heme ABC transporter	7.71E-08	−2.72	↓	↑
_01081	2	heme transporter IsdA	2.63E-07	−2.11	↓	↑
_01136	2	anti protein (phenol soluble modulin)	3.38E-07	−2.57	↓	↑
_01121	2	alpha-hemolysin precursor	4.12E-07	−2.07	↓	↑
_02696	2	fmhA protein, putative	4.40E-07	−2.29	↓	↑
_01082	2	heme transporter IsdC	1.40E-06	−2.81	↓	↑
_00354	2	putative enterotoxin	1.43E-05	3.22	↑	↓
_02740	2	drug transporter, putative	0.000142	1.65	↑	↓
_02129	2	staphostatin A	0.000392	−2.16	↓	↑
_00395	2	homology to known superantigen proteins	0.000545	2.81	↑	↓
_02718	2	ABC Transporters	0.000631	−2.09	↓	↑
_01932	2	type I restriction-modification enzyme, S subunit, EcoA family, putative	0.000692	2.06	↑	↓
_00998	2	fmt protein, putative	0.00072	1.59	↑	↓
_00261	2	type VII secretion protein EssB	0.000785	−1.84	↓	↑
_00426	2	ABC transporter, substrate-binding protein, putative	0.001147	−1.74	↓	↑
_00397	2	type I restriction-modification system, M subunit	0.002795	1.56	↑	↓
_02719	2	methicillin resistance protein FmtB	0.00383	−2.08	↓	↑
_00249	2	ABC-2 type transport system ATP-binding protein	0.005426	−1.83	↓	↑
_01448	3	Permeases of the major facilitator superfamily	1.82E-41	−5.16	↓	↑
_02420	3	Permeases of the major facilitator superfamily	2.58E-11	3.99	↑	↓
_01085	3	multidrug resistance protein SepA	1.17E-08	−3.16	↓	↑
_02687	3	ABC-type Fe3 + -hydroxamate transport system, periplasmic component	8.11E-08	2.50	↑	↓
_02435	3	formate/nitrite transporter, putative	2.63E-07	−2.42	↓	↑
_01087	3	Permeases of the major facilitator superfamily	9.18E-07	−3.16	↓	↑
_01086	3	iron compound ABC transporter, permease protein	9.72E-07	−2.74	↓	↑
_00105	3	iron compound ABC transporter, permease protein, putative	1.76E-06	−2.81	↓	↑
_00074	3	phosphonate ABC transporter, substrate-binding protein, putative	2.13E-06	−1.95	↓	↑
_02864	3	periplasmic binding protein, putative	1.36E-05	−2.13	↓	↑
_02766	3	ferrous iron transport protein B	3.06E-05	−1.95	↓	↑
_02763	3	peptide ABC transporter, permease protein, putative	5.33E-05	−1.97	↓	↑
_00423	3	peptide ABC transporter, ATP-binding protein, putative	0.000135	−2.30	↓	↑
_02740	3	ABC-type metal ion transport system, ATPase component	0.000142	1.65	↑	↓
_01893	3	drug transporter, putative	0.000208	−2.53	↓	↑
_02765	3	arsenical pump membrane protein subfamily	0.000471	−1.75	↓	↑
_00424	3	nickel ABC transporter, permease protein, putative	0.000582	−2.47	↓	↑
_02764	3	ABC transporter, permease protein, putative	0.000687	−1.70	↓	↑
_00325	3	peptide ABC transporter, ATP-binding protein, putative	0.000864	−2.44	↓	↑
_00104	3	Predicted periplasmic lipoprotein involved in iron transport	0.001514	−2.10	↓	↑
_00703	3	amino acid ABC transporter, ATP-binding protein, putative	0.002597	1.67	↑	↓
_02865	3	quinolone resistance norA protein, putative	0.004758	−2.19	↓	↑
_00102	3	ferrous iron transport protein A	0.00479	−1.91	↓	↑
_00103	3	phosphonates ABC transporter, permease protein CC0363, putative	0.008185	−1.78	↓	↑

For each Locus Tag, Function: 1) Amino acid transport and metabolism, 2) Defence mechanisms, and 3) Inorganic ion transport and metabolism; Protein Description, *P*-value adjusted (padj), log2FoldChange (log2FC) and (↑) up and (↓) down-regulation were reported

### Virulence and defence pathways analysis: comparison between genomic and transcriptomic data

Functional and pathway enrichment analysis for integrated regulatory network of the two groups of strains was performed considering annotated genes from DNA-Seq and RNA-Seq experiments, grouped in the three dataset, i.e. gene exclusively present in GTB/ST8ra, gene exclusively present in GTS/ST398ra and DEGs (Additional files 5 and 8).

**Fig. 4:**
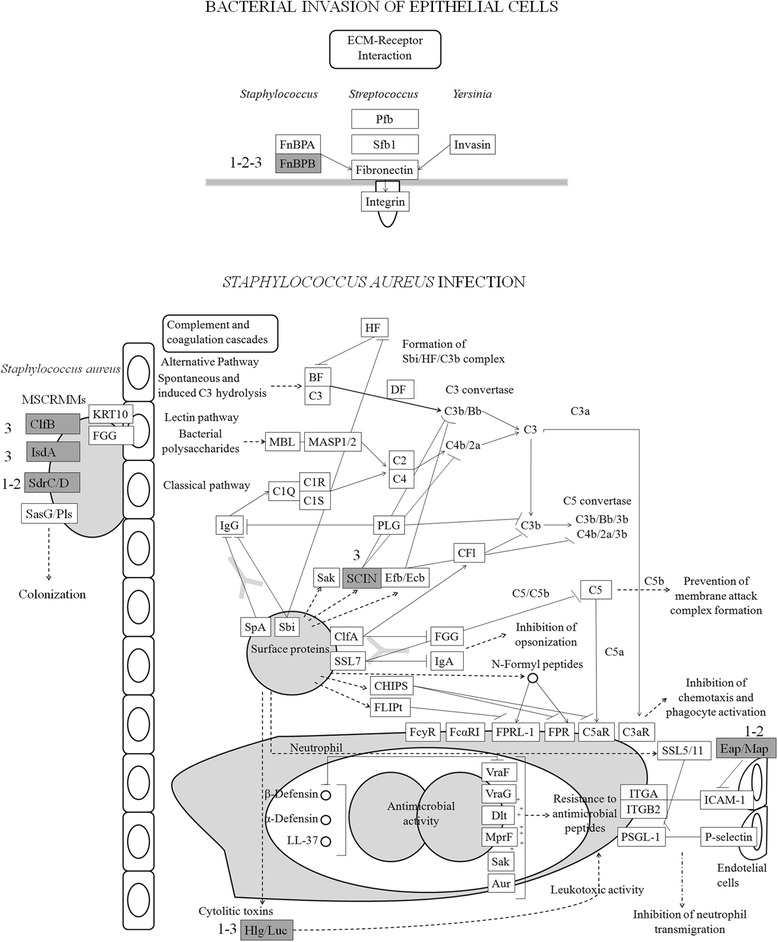
Bacterial invasion of epithelial cells and infection pathways analysis of *Staph. aureus*. In grey highlighted DEGs or unique genes for GTB/ST8ra or GTS/ST398ra. Fibronectin-Binding Protein B *FnBPB*, clumping factor B *ClfB*, iron-regulated surface determinant protein A *IsdA*, serine-aspartate repeat-containing protein C/D/E *Sdr*C/D/E, staphylococcal complement inhibitor (SCIN) and *eap/map* protein *EaP*/*Map* and leukocidin/hemolysin Hlg/Luc. Numbers define the dataset from which the genes were found: 1) unique GTB/ST8ra, 2) unique GTS/ST398ra and 3) DEGs from transcriptomic analysis, and their combination: (1-2: genes with differences in protein sequence; 1-3: genes expressed and present only in GTBra; 1-2-3: genes differentially expressed and different in protein sequence between the two reference genotypes)

Considering the functional categories belonging to bacterial invasion of epithelial cells and infection pathways, we found genes that were detected by using or (i) strain genotyping comparison or (ii) transcriptomic analysis or (iii) by the two combined approaches, as reported in Fig. 4. For example, from this analysis Fibronectin-Binding Protein B gene (*fnbB),* an important adhesin involved not only in adhesion to cells but also in internalization by cells, was detected in both groups by means of a partial alignment between GTB/ST8ra and GTS/ST398ra protein sequence. In parallel, transcriptomic analysis revealed a variation in *fnbB* expression, over-expressed in GTB/ST8 and down regulated in GTS/ST398 (Additional file 10 a, b, c). The nucleotide sequence between the two groups of strains was verified by sequencing a 1285 bp portion of *fnbB* gene (Additional file 11). The nucleotide sequence was conserved within each group of strains and was high polymorphic between the two genotypes. Overall, GTS/ST398 showed a high polymorphism compared to reference NCTC8325 strain sequence (83% of identity), whereas no differences were observed for GTB/ST8 (100% of identity). Further, the protein translation for GTS/ST398 resulted in a truncated protein form.

Moreover as shown in Fig. 4, clumping factor B gene (*ClfB*) and iron-regulated surface determinant protein A gene (*IsdA)* showed differentially expression by transcriptomic profiling (over-expressed and down regulated in GTB/ST8 and vice versa for GTS/ST398, respectively) (Additional file 12 a, b). Furthermore, the serine-aspartate repeat-containing protein C/D/E gene (*SdrC/D/E)* and *eap*/*map* protein gene (*EaP/MaP)* differed in protein sequence exclusively by DNASeq comparison (Additional file 13 a, b). Finally leukocidin/hemolysin gene (*Hlg/Luc*) was seen to be exclusively present and expressed in the GTB/ST8 strains, whereas the staphylococcal complement inhibitor SCIN (*scn*) was differentially expressed between the two groups, over-expressed in GTB/ST8 and down-regulated in GTS/ST398.

## Discussion

*Staph. aureus* IMI clinical outcomes are highly variable and depend on several factors, including animal genetics, environmental conditions and strain-dependent factors. All these conditions should be correctly evaluated in order to predict the spread of bacterial strains within the herd. Here we achieved an in-depth characterization through NGS of six *Staph. aureus* strains previously genotyped as GTB/ST8 and GTS/ST398, differing in virulence properties such as within-herd *Staph. aureus* IMI prevalence [4].

These six strains belonged to two distinct clonal complexes and sequence types (CC8/ST8 for GTB/ST8, and CC398/ST398 for GTS/ST398), with a high intra-group similarity among the three strains associated to the same clonal complex (about 97% and 95% for GTB/ST8 and GTS/ST398, respectively), significantly supporting the creation of the reference assemblies as representative of these two lineages. Both genotypes clusterized in groups that include strains of human origin: GTS/ST398 showed high similarity with the prototype of *Staph. aureus* ST398, SO385, isolated from human endocarditis [49], whereas GTB/ST8 to *Staph. aureus* TCH1516, a methicillin susceptible ST8 strain, isolated from an adolescent patient with severe sepsis syndrome [50]. Recent studies on these CCs [4, 13] showed a closed genetic relationship between CC8 isolated from dairy cow mastitis and human CC8, suggesting human-to-bovine jump. On the other hand, the presence of CC398 strains was described only in herds with IMI prevalence lower than 5% [4]. In the present study, a comparison of the genome sequences of these strains with a core genome (set of genes shared between GTB/ST8ra and GTS/ST398ra) revealed about 17% of differences for their gene content, with a relevant enrichment in genes associated with virulence properties. Both groups of strains showed differences in several genes associated to virulence factors and some of them were present in only one of the two genotypes.

Furthermore, the transcriptomic profiling for both groups of strains confirmed the functional enrichment for genes related to adaptation and propensity to chronicity. Interestingly, GTB/ST8ra showed higher expression of signal transduction Target of RNAIII-activating Protein TRAP, that leads to the activation of *agr* system, resulting in the expression of several virulence factors. As previously described [51, 52], the protein TRAP activates RNAIII synthesis by RNAIII-activating protein (RAP) system, increasing the pathogenic potential of the bacteria.

Contemporary, the integrated pathway analysis between the two genotypes of *Staph. aureus* genes involved in pathogenicity showed an interesting variation in the microbial surface component recognizing adhesive matrix molecule (MSCRAMM), whose function includes adhesion to and invasion in host cells and tissues, evasion of immune responses and biofilm formation [53]. As well known, the *fnb*B gene is a multifunctional MSCRAMM, which recognizes fibronectin, fibrinogen and elastin and promoting the internalization of *Staph. aureus* into epithelial and endothelial cell mediating bacterial invasion [54, 55]. Most *Staph. aureus* strains can express two distinct fibronectin-binding proteins (FnBPA and FnBPB), which both mediate adhesion to fibrinogen, elastin and fibronectin. The GTB/ST8 and GTS/ST398 strains, analysed in this study, presented the two fibronectin-binding proteins but only *fnb*B showed changes in genomic and transcriptomic analyses between these two groups. The *fnb*B gene showed high variability between the two genotypes, revealing a high level of polymorphisms that lead to a premature stop codon and a truncated form of the protein for GTS/ST398 strains. Similar results were previously published by McCarthy and colleagues [56], which postulated that the truncated FnBPB form could affect *Staph. aureus* colonisation and infection. Also Burke and co-workers found different FnBPB isotypes in diverse STs *Staph. aureus* strains, revealing an association between this gene and the invasiveness [57]. And more, the presence of protein variations for both groups of strains suggests a different affinity for fibronectin, necessary for the internalization of *Staph. aureus* into host cells [23].

In addition, *ClfB* and *SdrC/D/E* genes encoding for MSCRAMMs proteins, whose functions are related to adhesion and colonization [58, 59], showed differences by transcriptomic and genomic analyses in both genotypes, respectively, indicating that surface adhesins are not only present/absent, but also variable amongst lineages of *Staph. aureus* [56]*,* such as GTB/ST8 and GTS/ST398. Polymorphisms in these genes are well known and used to Multilocus Variable Number Tandem Repeat Fingerprinting (MLVF), a genotyping method for epidemiological studies [60]. Other multi-functional proteins, such as the “*Staph. aureus* surface G Sas G protein the iron-dependent adhesion IsdA, IsdB, IsdC”, over-expressed in GTS/ST398, play a role in biofilm formation [61, 62]. The adhesive properties displayed by MSCRAMM proteins reside within the cell surface; however, several important adhesins are also formally secreted from the bacterial cell. The “Secreted Expanded Repertoire Adhesive Molecules (SERAM) extracellular adherence protein” (*eap*) is nearly ubiquitously distributed amongst *Staph. aureus* strains and appears to function as a virulence determinant in animal models of chronic infection [63]. *Eap* gene was proposed as novel target for specific identification of *Staph. aureus* [64]. According to our results the sequence alignment of *eap* gene from all *Staph. aureus* genomes published to date revealed a significant polymorphism in this gene [65]. As stated by McCarthy et al. [56], the genetic variation in *Staph. aureus* surface and immune evasion genes is lineage associated and carries a range of unique variants in order to improve the adaptation of this microorganism to different host species.

Finally, the staphylococcal complement inhibitor (*scn*), over-expressed in GTB/ST8 and down-regulated in GTS/ST398, produced by the *Staph. aureus* during the early phase of infection, helped the microorganism to survive into the host [66], preventing both chemotaxis and phagocytosis. The fact that surface and immune evasion proteins are different between lineages suggests that they are essential for virulence, opening a window for further investigations.

## Conclusions

In conclusion, our analysis proves that integration of RNA-Seq and DNA-Seq data well depicts *Staph. aureus* strains associated with different within-herd IMI prevalence in dairy cows. Our results disclosed congruent patterns of genetic variation in colonization and invasion factors between GTB/ST8 and GTS/ST398 strains. Notwithstanding, results highlight a high number of unknown genes differing between genotypes, whose unknown functionality lacks a direct association with virulence function. Overall, the fine genomic characterization of these strains was a first step towards developing strategies able to provide new insights into mechanisms associated to *Staph. aureus* mastitis, including genomic comparison of a larger set of high and low diffusive strains, improvement of *Staph. aureus* reference strains annotation and new *ad hoc* bioinformatic tools.

## Additional files

Additional file 1: GTS/ST398 and GTB/ST8 reads cross-mapped against NCTC 8325 genome and selected and used in RNASeq analysis for quantification and differential expression analysis. (XLSX 183 kb)
Additional file 2: List of the 22 *Staph. aureus* strains available in NCBI used in this study for genomic comparative analysis. (DOCX 22 kb)
Additional file 3: Primer list used for Real-Time-PCR experiments. (DOCX 17 kb)
Additional file 4: Sequencing results for three GTB/ST8 and three GTS/ST398 strains. A) Assembly statistics for each strain: number of total contig obtained (N° of contigs), number of contig bigger that 500 nt in size (N° of contigs > 500), max length of the contig (Max contig length), median of contig lengths (N50), total genome assembled (Total assembled) were reported. B) Sequencing mapping GTS/ST398 vs GTB/ST8 and NCTC8325 statistics for each strain: number of total reads (Raw reads), number of reads mapped on NCTC8325 reference (Mapping reads), the percentage (Mapping rate (%)), the average sequencing depth (Mean Depth (fold)) and the percentage of genome reference coverage (Coverage (%)) were reported. C) Sequencing mapping GTB/ST8 vs GTS/ST398 and NCTC8325 statistics for each strain: number of total reads (Raw reads), number of reads mapped on NCTC8325 reference (Mapping reads), the percentage (Mapping rate (%)), the average sequencing depth (Mean Depth (fold)) and the percentage of genome reference coverage (Coverage (%)) were reported. (DOCX 39 kb)
Additional file 5: List of shared and unique genes for single reference sequence representing genotype GTB/ST8ra and GTS/ST398ra. The gene annotation (Category, Subcategory, Subsystem, Role) was reported for each entry. (XLS 762 kb)
Additional file 6: Functional enrichment analysis of COGs distributions calculated by using the Fisher’s test and Bonferroni multiple testing correction for GTB/ST8ra and GTS/ST398ra. (XLSX 17 kb)
Additional file 7: RNA sequencing results for the three GTB/ST8 and th three GTS/ST398 strains. Mapping vs NCTC8325 statistics for each strain: number of total reads (Raw reads), number of reads mapped on NCTC8325 reference (Maping reads) and percentage (% Mapping), total CDS detected (CDS detected) and percentage of CDS vs reference CDS (%CDS detected), distribution of CDS detected on mRNA (% mRNA), rRNA (% rRNA) and intergenic region (% Intergenic). (DOCX 30 kb)
Additional file 8: Differential expressed genes (DEGs) between the three GTB/ST8 and the three GTS/ST398 strains. GTS/ST398 (1_2_3)_all CDS, GTB/ST8 (1_2_3)_all CDS, log2FoldChange, padj, protein and function class description were reported for each locus tag. In bold gene related to defense mechanism in italic gene validate by Real Time PCR experiment. (XLSX 33 kb)
Additional file 9: a) Real Time Fold Change variation and b) Fold change average between two reference genotypes GTB-ST8 and GTS-ST398 for three selected genes: SAOUHSC_00773, SAOUHSC_01181, SAOUHSC_01450. (DOC 34 kb)
Additional file 10: Fibronectin-Binding Protein B FnBpB comparison between *Staph*. *aureus* GTB/ST8ra and GTS/ST398ra. a) FnBpB protein sequence for GTB/ST8ra (>fig|6666666.84847.peg.316 and GTS/ST398ra (>fig|6666666.84857.peg.2046, >fig|6666666.84857.peg.2047) b) Protein blast between GTB/ST8ra and GTS/ST398ra. c) Integrative Genomics Viewer (IGV) view comparison between three *Staph*. GTB/ST8 and three GTS/ST398 strains mapped on *Staph. aureus* NCTC 8325 strain with reads from DNASeq or RNA-Seq experiments. (DOCX 176 kb)
Additional file 11: Sequence alignment for a 1285 bp portion of *fnbB* gene (from position 1297up to 2582; NCBI accession number: CP000253, region: 2577879.....2580632) in all the six strains. In figure, gene sequence for GTS/ST398 (1-2-3), GTB/ST8 (1-2-3), and the NCTC8325 strain in the homologous position are presented. (DOCX 670 kb)
Additional file 12: Integrative Genomics Viewer (IGV) view comparison of a) clumping factor B *Clf*B and b) protein A IsdA, between *Staph. aureus* three GTB/ST8 and three GTS/ST398 strains mapped on *Staph. aureus* NCTC 8325 strain with reads from DNASeq or RNASeq experiment. (DOCX 316 kb)
Additional file 13: Serine-aspartate repeat-containing protein C/D/E SdrC/D/E and eap, map protein EaP/MaP comparison between *Staph. aureus* GTB/ST8ra and GTS/ST398ra. a) SdrC/D/E protein sequenze for GTB/ST8ra (>fig|6666666.84847.peg.2863) and GTS/ST398ra (>fig|6666666.84857.peg.510); Eap/Map protein sequence for GTB/ST8ra (>fig|6666666.84847.peg.1290) and GTS/ST398ra (>fig|6666666.84857.peg.609). b) Protein blast for SdrC/D/E and Eap/Map between GTB/ST8ra and GTS/ST398ra. (DOCX 42 kb)

## Abbreviations

BHI: Brain heart infusion
CDS: Coding DNA sequence
DNA-Seq: DNA sequencing
GTB: Genotype B
GTS: Genotype S
IMI: Intra-mammary infection
MLST: Multi locus sequence type
NGS: Next generation sequencing
RNA-Seq: RNA sequencing
RS-PCR: Repetitive-sequence PCR
RT-PCR: Real time PCR
ST: Sequence type
*Staph. aureus*: *Staphylococcus aureus*
