# Supplementary material for: Genomic and transcriptomic comparison between Staphylococcus aureus strains associated with high and low within herd prevalence of intra-mammary infection
Source: BMC Microbiol. 2017 Jan 19;17:21. doi: 10.1186/s12866-017-0931-8 (PMC5247818; doi:10.1186/s12866-017-0931-8)
Supplement: Additional file 2: — List of the 22 Staph. aureus strains available in NCBI used in this study for genomic comparative analysis. (DOCX 22 kb) [file 12866_2017_931_MOESM2_ESM.docx]

| Strain | Accession Number |
| --- | --- |
| *Staph. aureus* NCTC8325 | CP000253 |
| *Staph. aureus* MSHR1132 | FR821777 |
| *Staph. aureus* ST398 isolate SO385 | AM990994 |
| *Staph. aureus* CN1 | CP003981 |
| *Staph. aureus* 11819-97 | CP003194 |
| *Staph. aureus* N315 | AP003139 |
| *Staph. aureus* Mu50 | AP003367 |
| *Staph. aureus* JH1 | CP000736 |
| *Staph. aureus* JH9 | CP000703 |
| *Staph. aureus* COL | CP000045 |
| *Staph. aureus* VC40 | CP003033 |
| *Staph. aureus* Newman | AP009351 |
| *Staph. aureus* USA300_TCH1516 | CP001544 |
| *Staph. aureus* USA300_FPR3757 | CP000255 |
| *Staph. aureus* MW2 | BA000033 |
| *Staph. aureus* Bmb9393 | CP005288 |
| *Staph. aureus* TW20 | FN433597 |
| *Staph. aureus* ED133 | CP001996 |
| *Staph. aureus* JKD6159 | CP002115 |
| *Staph. aureus* CC45 | CP006045 |
| *Staph. aureus* MRSA252 | BX571856 |
| *Staph. aureus* TCH60 | CP002111 |

Additional file 2. List of the 22 *Staph. aureus* strains available in NCBI used in this study for genomic comparative analysis.
