# Supplementary material for: Genomic and transcriptomic comparison between Staphylococcus aureus strains associated with high and low within herd prevalence of intra-mammary infection
Source: BMC Microbiol. 2017 Jan 19;17:21. doi: 10.1186/s12866-017-0931-8 (PMC5247818; doi:10.1186/s12866-017-0931-8)
Supplement: Additional file 3: — Primer list used for Real-Time-PCR experiments. (DOCX 17 kb) [file 12866_2017_931_MOESM3_ESM.docx]

**Additional file 3**. Primer used for Real time PCR

| **GENES or Locus Tag** | **FORWARD** | **REVERSE** |
| --- | --- | --- |
| proC | TGCCAAAATCCAGTTGCTAGAA | GCCAGTAACAGAGTGTCCAACTTG |
| SAOUHSC_00773 | ACCAGAAGTTGGCGCTATTTTC | TTCAACAACACCAACATGTCCAT |
| SAOUHSC_01181 | CCTTGTTCATCAGCAGGTTTGA | CAGGTATCCCACACATTTTAGCAA |
| SAOUHSC_01314 | TCGATAGTGATTGTGAGGTGAAAAGT | TTGAGTCGACTGCTAAAGTTCCAT |
| SAOUHSC_01450 | CAGGGATTGCAGCAGCAA | GGCGGCATCATTACCATTTG |
