# Supplementary material for: Genomic and transcriptomic comparison between Staphylococcus aureus strains associated with high and low within herd prevalence of intra-mammary infection
Source: BMC Microbiol. 2017 Jan 19;17:21. doi: 10.1186/s12866-017-0931-8 (PMC5247818; doi:10.1186/s12866-017-0931-8)
Supplement: Additional file 4: — Sequencing results for three GTB/ST8 and three GTS/ST398 strains. A) Assembly statistics for each strain: number of total contig obtained (N° of contigs), number of contig bigger that 500 nt in size (N° of contigs > 500), max length of the contig (Max contig length), median of contig lengths (N50), total genome assembled (Total assembled) were reported. B) Sequencing mapping GTS/ST398 vs GTB/ST8 and NCTC8325 statistics for each strain: number of total reads (Raw reads), number of reads mapped on NCTC8325 reference (Mapping reads), the percentage (Mapping rate (%)), the average sequencing depth (Mean Depth (fold)) and the percentage of genome reference coverage (Coverage (%)) were reported. C) Sequencing mapping GTB/ST8 vs GTS/ST398 and NCTC8325 statistics for each strain: number of total reads (Raw reads), number of reads mapped on NCTC8325 reference (Mapping reads), the percentage (Mapping rate (%)), the average sequencing depth (Mean Depth (fold)) and the percentage of genome reference coverage (Coverage (%)) were reported. (DOCX 39 kb) [file 12866_2017_931_MOESM4_ESM.docx]

| **A) Sequencing Assembly** | | | | | | | | | |
| --- | --- | --- | --- | --- | --- | --- | --- | --- | --- |
|  | **GTS/ST398** | | | | | **GTB/ST8** | | | |
|  | **Strain 1** | | **Strain 2** | **Strain 3** | | **Strain 1** | **Strain 2** | | **Strain 3** |
| N° of contigs | 606 | | 280 | 585 | | 429 | 529 | | 168 |
| N° of contigs> 500 | 105 | | 66 | 248 | | 43 | 72 | | 36 |
| Max contig length | 408,608 | | 681,543 | 296,697 | | 1,058,718 | 893,892 | | 1,024,497 |
| N50 | 233,663 | | 419,910 | 126,075 | | 984,749 | 564,734 | | 982,047 |
| Total assembled | 2,785,759 | | 2,765,923 | 2,905,478 | | 2,803,645 | 2,848,501 | | 2,764,543 |
|  | | | | | | | | | |
| **B)   Sequencing mapping GTS/ST398** | | | | | | | |  |  |
|  | | **GTB/ST8** | | | **NCTC8325** | | |  |  |
| Raw reads | | 8.128.972 | | | 8.128.972 | | |  |  |
| Mapping reads | | 5.705.062 | | | 6.965.450 | | |  |  |
| Mapping rate (%) | | 70.18% | | | 85.68% | | |  |  |
| Mean Depth (fold) | | 630.47 | | | 761.77 | | |  |  |
| Coverage (%) | | 69.36% | | | 95.15% | | |  |  |
| **C)   Sequencing mapping vs GTB/ST8** | | | | | | | |  |  |
|  | | **GTS/ST398** | | | **NCTC8325** | | |  |  |
| Raw reads | | 9.087.346 | | | 9.087.346 | | |  |  |
| Mapping reads | | 7.566.439 | | | 8.399.955 | | |  |  |
| Mapping rate (%) | | 83.26% | | | 92.43% | | |  |  |
| Mean Depth (fold) | | 735.26 | | | 888.31 | | |  |  |
| Coverage (%) | | 80.02% | | | 97.46% | | |  |  |

**Additional File 4.**Sequencing results for 3 GTB/ST8 and 3 GTS/ST398 strains. A) Assemblystatistics for each strain: number of total contig obtained (N° of contigs), number of contig bigger that 500 nt in size (N° of contigs> 500), max length of the contig (Max contig length), median of contig lengths (N50), total genome assembled (Total assembled) were reported.The second table contains B) GTS/ST398 genome sequencing mapping vs GTB/ST8 and NCTC8325and C) GTB/ST8 genome sequencing mappingvsGTS/ST398 and NCTC8325statistics: number of total reads (Raw reads), number of reads mapped on NCTC8325 reference (Mapping reads), the percentage (Mapping rate (%)), the average sequencing depth (Mean Depth (fold)) and the percentage of genome reference coverage (Coverage (%)) were reported.
