# Supplementary material for: Genomic and transcriptomic comparison between Staphylococcus aureus strains associated with high and low within herd prevalence of intra-mammary infection
Source: BMC Microbiol. 2017 Jan 19;17:21. doi: 10.1186/s12866-017-0931-8 (PMC5247818; doi:10.1186/s12866-017-0931-8)
Supplement: Additional file 7: — RNA sequencing results for the three GTB/ST8 and th three GTS/ST398 strains. Mapping vs NCTC8325 statistics for each strain: number of total reads (Raw reads), number of reads mapped on NCTC8325 reference (Maping reads) and percentage (% Mapping), total CDS detected (CDS detected) and percentage of CDS vs reference CDS (%CDS detected), distribution of CDS detected on mRNA (% mRNA), rRNA (% rRNA) and intergenic region (% Intergenic). (DOCX 30 kb) [file 12866_2017_931_MOESM7_ESM.docx]

|  | **GTS/ST398** | | | **GTB/ST8** | | |
| --- | --- | --- | --- | --- | --- | --- |
|  | **Strain 1** | **Strain 2** | **Strain 3** | **Strain 1** | **Strain 2** | **Strain 3** |
| Raw reads | 3,026,972 | 3,370,420 | 2,241,480 | 2,933,330 | 2,458,170 | 2,594,786 |
| Mapping reads | 2,827,855 | 3,113,259 | 2,056,531 | 2,717,298 | 2,247,906 | 2,479,609 |
| % Mapping | 93.42 | 92.37 | 91.75 | 92.64 | 91.45 | 95.56 |
| **CDS mapping** |  |  |  |  |  |  |
| %mRNA | 91.56 | 89.22 | 83.04 | 87.34 | 86.25 | 90.76 |
| %rRNA | 0.30 | 0.38 | 5.20 | 5.20 | 4.87 | 0.86 |
| % Intergenic | 8.13 | 10.39 | 15.57 | 7.45 | 8.86 | 8.36 |

**Additional File 7.** RNA sequencing results for 3 GTB/ST8 and 3 GTS/ST398 strains. Mapping vs NCTC8325 statistics for each strain: number of total reads (Raw reads), number of reads mapped on NCTC8325 reference (Maping reads) and percentage (%Mapping), distribution of CDS detected in the core genome on mRNA (% mRNA), rRNA (% rRNA ) and intergenic region (% Intergenic).
