# Supplementary material for: Genomic and transcriptomic comparison between Staphylococcus aureus strains associated with high and low within herd prevalence of intra-mammary infection
Source: BMC Microbiol. 2017 Jan 19;17:21. doi: 10.1186/s12866-017-0931-8 (PMC5247818; doi:10.1186/s12866-017-0931-8)
Supplement: Additional file 9: — a) Real Time Fold Change variation and b) Fold change average between two reference genotypes GTB-ST8 and GTS-ST398 for three selected genes: SAOUHSC_00773, SAOUHSC_01181, SAOUHSC_01450. (DOC 34 kb) [file 12866_2017_931_MOESM9_ESM.doc]

9a)


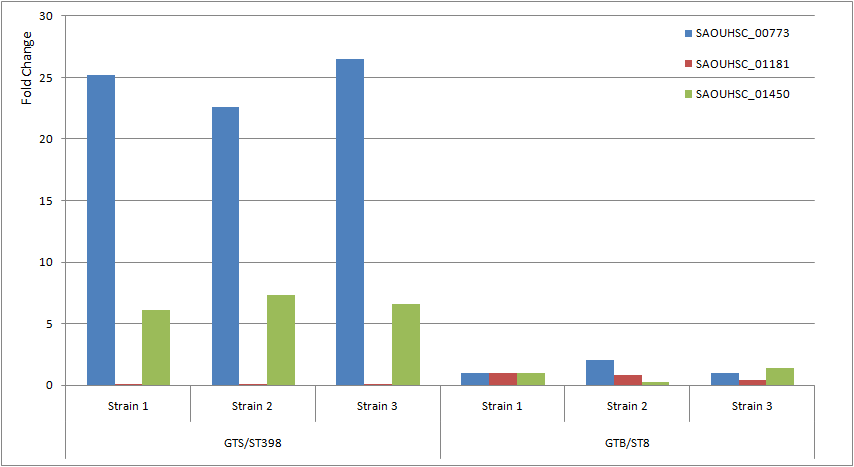


9b)

|  | **SAOUHSC_00773** | **SAOUHSC_01181** | **SAOUHSC_01450** | |
| --- | --- | --- | --- | --- |
| average **GTB-ST8**/ average **GTS-ST398** | 0,053780378 | 9,097167413 | 0,129697385 |  |

**Additional File 9.** a) Real Time Fold Change variation and b) Fold change average between two reference genotypes GTB-ST8 and GTS-ST398 for three selected genes: SAOUHSC_00773, SAOUHSC_01181, SAOUHSC_01450.
