# Supplementary material for: Genomic and transcriptomic comparison between Staphylococcus aureus strains associated with high and low within herd prevalence of intra-mammary infection
Source: BMC Microbiol. 2017 Jan 19;17:21. doi: 10.1186/s12866-017-0931-8 (PMC5247818; doi:10.1186/s12866-017-0931-8)

**Additional file 10a**.

**Sequence FnBpB Genotype GTB/ST8ra**

>fig|6666666.84847.peg.316

MIVVGMGQEKEAAASEQNNTTVEESGSSATESKASETQTTTNNVNTIDETQSYSATSTEQPSQSTQVTTEEAPKTVQAPKVETSRVDLPSEKVADKETTGTQVDIAQPSNVSEIKPRMKRSTDVTAVAEKEVVEETKATGTDVTNKVEVEEGSEIVGHKQDTNVVNPHNAERVTLKYKWKFGEGIKAGDYFDFTLSDNVETHGISTLRKVPEIKSTDGQVMATGEIIGERKVRYTFKEYVQEKKDLTAELSLNLFIDPTTVTQKGNQNVEVKLGETTVSKIFNIQYLGGVRDNWGVTANGRIDTLNKVDGKFSHFAYMKPNNQSLSSVTVTGQVTKGNKPGVNNPTVKVYKHIGSDDLAESVYAKLDDVSKFEDVTDNMSLDFDTNGGYSLNFNNLDQSKNYVIKYEGYYDSNASNLEFQTHLFGYYNYYYTSNLTWKNGVAFYSNNAQGDGKDKLKEPIIEHSTPIELEFKSEPPVEKHELTGTIEESNDSKPIDFEYHTAVEGAEGHAEGTIETEEDSIHVDFEESTHENSKHHADVVEYEEDTNPGGGQVTTESNLVEFDEDSTKGIVTGAVSDHTTIEDTKEYTTESNLIELVDELPEEHGQAQGPIEEITENNHHISHSGLGTENGHGNYGVIEEIEENSHVDIKSELGYEGGQNSGNQSFEEDTEEDKPKYEQGGNIVDIDFDSVPQIHGQNNGNQSFEEDTEKDKPKYEQGGNIIDIDFDSVPHIHGFNKHTEIIEEDTNKDKPNYQFGGHNSVDFEEDTLPQVSGHNEGQQTIEEDTTPPIVPPTPPTPEVPSEPETPTPPTPEVPTEPGKPIPPAKEEPKKPSKPVEQGKVVTPVIEINEKVKAVVPTKKAQSKKSELPETGGEESTNNGMLFGGLFSILGLALLRRNKKNHKA

**Sequence FnBpB Genotype GTS/ST398ra**

>fig|6666666.84857.peg.2046

MKSNLRYGIRKHKLGAASVFLGTMIVVGMGQEKEAAASEQNNTTVEESGNSATESKASETQTTTNNVNTIDETQSYSATSTEQPSQSTQVTTEEAPTTVQAPKAETSRVDLPSEKAADKGIVGTQVDTTQPSNVSEIKPRMKRSADVTAASEKEVVEEAKVTGTDVTSKVKVEEGSEIVGHNNKETNVVNPHNAERVTLKYKWKFEDGIKPGDYFDFTLSNNVETHGISPLRKVPDIKSKDGQVMAVGEVNEERKIRYTFKEYVQGKKDLKAELDLNLFIDPTTVTKRGNQNIEVTLGEKKISKQFDIKYLDGVKDNWGVTVNGRIHTLNKQEGRFSHFAYVKPNNQSLTSVTVTGQVTSGYKQNAKNPTV

>fig|6666666.84857.peg.2047

MTGTVEESDDSKPIDFEYHTAVEGAEGHAEGTIETEEDSIHVDFEESTHENSKHHADVVEYEEDTNPGGGQVTTESNLVEFDEESTKGIVTGAVSDHTTVEDTKEYTTESNLIELVDELPEEHGQAQGPIEEITENNHHISHSGLGTENGHGNYGVIDEIEENSHVDIKSELGYEGGQNSGNQSFEEDTEEDKPKYEQGGNIVDIDFDSVPQIHGQNNGNQSFEEDTEEDKPKYEQGGNIIDIDFDSVPQIHGFNKHNEIIEEDTNKDKPNYQFGGHNSVDFEEDTLPKVSGQNEGQQTIEEDTTPPTPEVPSEPETPTPPTPEVPSEPGEPTPPTPEVPSEPETPVPPTPEVPSEPGKPVPPAKEEPKKPSKPVEQGKVVTPVIEINEKVKAVAPTKQKQSKKSELPETGGEESTNKGILFGGLFSILGLALLRRNKKNHKA

**Additional file 10b**.

**Blast >fig|6666666.84847.peg.316 (GTB/ST8ra) vs >fig|6666666.84857.peg.2046 (GTS/ST398ra)**

| \|  \| 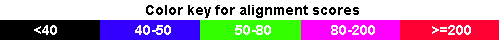 \| \| --- \| --- \|  \| 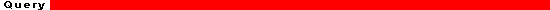 \| \| --- \|  \| 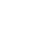 \| 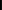 \| 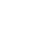 \| 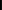 \| 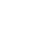 \| 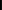 \| 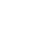 \| 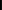 \| 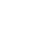 \| 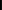 \| 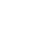 \| 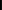 \| 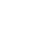 \| 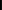 \| \| --- \| --- \| --- \| --- \| --- \| --- \| --- \| --- \| --- \| --- \| --- \| --- \| --- \| --- \|  \| 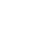 \| 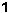 \| 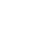 \| 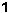 \| 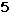 \| 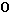 \| 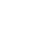 \| 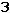 \| 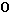 \| 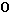 \| 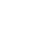 \| 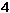 \| 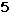 \| 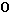 \| 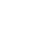 \| 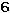 \| 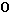 \| 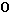 \| 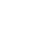 \| 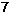 \| 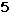 \| 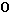 \| 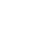 \| 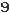 \| 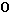 \| 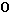 \| \| --- \| --- \| --- \| --- \| --- \| --- \| --- \| --- \| --- \| --- \| --- \| --- \| --- \| --- \| --- \| --- \| --- \| --- \| --- \| --- \| --- \| --- \| --- \| --- \| --- \| --- \|  \| 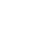 \| [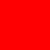](http://blast.ncbi.nlm.nih.gov/Blast.cgi#Query_188121) \| \| --- \| --- \|  \| 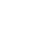 \| \| --- \| | | | | | |
| --- | --- | --- | --- | --- | --- | --- | --- | --- | --- | --- | --- | --- | --- | --- | --- | --- | --- | --- | --- | --- | --- | --- | --- | --- | --- | --- | --- | --- | --- | --- | --- | --- | --- | --- | --- | --- | --- | --- | --- | --- | --- | --- | --- | --- | --- | --- | --- | --- | --- | --- | --- |
| **Score** | **Expect** | **Method** | **Identities** | **Positives** | **Gaps** |
| 548 bits(1412) | 0.0 | Compositional matrix adjust. | 288/348(83%) | 310/348(89%) | 1/348(0%) |

Query 1 MIVVGMGQEKEAAASEQNNTTVEESGSSATESKASETQTTTNNVNTIDETQSYSATSTEQ 60

MIVVGMGQEKEAAASEQNNTTVEESG+SATESKASETQTTTNNVNTIDETQSYSATSTEQ

Sbjct 24 MIVVGMGQEKEAAASEQNNTTVEESGNSATESKASETQTTTNNVNTIDETQSYSATSTEQ 83

Query 61 PSQSTQVTTEEAPKTVQAPKVETSRVDLPSEKVADKETTGTQVDIAQPSNVSEIKPRMKR 120

PSQSTQVTTEEAP TVQAPK ETSRVDLPSEK ADK GTQVD QPSNVSEIKPRMKR

Sbjct 84 PSQSTQVTTEEAPTTVQAPKAETSRVDLPSEKAADKGIVGTQVDTTQPSNVSEIKPRMKR 143

Query 121 STDVTAVAEKEVVEETKATGTDVTNKVEVEEGSEIVGHK-QDTNVVNPHNAERVTLKYKW 179

S DVTA +EKEVVEE K TGTDVT+KV+VEEGSEIVGH ++TNVVNPHNAERVTLKYKW

Sbjct 144 SADVTAASEKEVVEEAKVTGTDVTSKVKVEEGSEIVGHNNKETNVVNPHNAERVTLKYKW 203

Query 180 KFGEGIKAGDYFDFTLSDNVETHGISTLRKVPEIKSTDGQVMATGEIIGERKVRYTFKEY 239

KF +GIK GDYFDFTLS+NVETHGIS LRKVP+IKS DGQVMA GE+ ERK+RYTFKEY

Sbjct 204 KFEDGIKPGDYFDFTLSNNVETHGISPLRKVPDIKSKDGQVMAVGEVNEERKIRYTFKEY 263

Query 240 VQEKKDLTAELSLNLFIDPTTVTQKGNQNVEVKLGETTVSKIFNIQYLGGVRDNWGVTAN 299

VQ KKDL AEL LNLFIDPTTVT++GNQN+EV LGE +SK F+I+YL GV+DNWGVT N

Sbjct 264 VQGKKDLKAELDLNLFIDPTTVTKRGNQNIEVTLGEKKISKQFDIKYLDGVKDNWGVTVN 323

Query 300 GRIDTLNKVDGKFSHFAYMKPNNQSLSSVTVTGQVTKGNKPGVNNPTV 347

GRI TLNK +G+FSHFAY+KPNNQSL+SVTVTGQVT G K NPTV

Sbjct 324 GRIHTLNKQEGRFSHFAYVKPNNQSLTSVTVTGQVTSGYKQNAKNPTV 371

**Blast >fig|6666666.84847.peg.316 (GTB/ST8ra) vs >fig|6666666.84857.peg.2047 (GTS/ST398ra)**

| \| 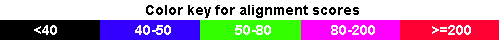 \| \| --- \|  \| 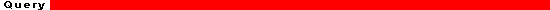 \| \| --- \|  \| 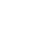 \| 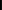 \| 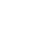 \| 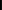 \| 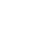 \| 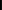 \| 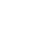 \| 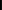 \| 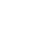 \| 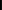 \| 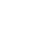 \| 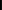 \| 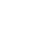 \| 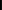 \| \| --- \| --- \| --- \| --- \| --- \| --- \| --- \| --- \| --- \| --- \| --- \| --- \| --- \| --- \|  \| 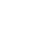 \| 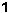 \| 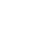 \| 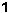 \| 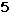 \| 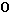 \| 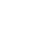 \| 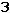 \| 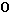 \| 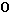 \| 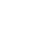 \| 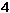 \| 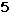 \| 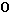 \| 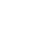 \| 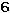 \| 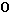 \| 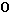 \| 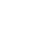 \| 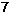 \| 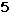 \| 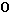 \| 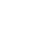 \| 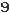 \| 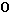 \| 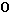 \| \| --- \| --- \| --- \| --- \| --- \| --- \| --- \| --- \| --- \| --- \| --- \| --- \| --- \| --- \| --- \| --- \| --- \| --- \| --- \| --- \| --- \| --- \| --- \| --- \| --- \| --- \|  \| 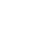 \| 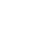 \| [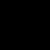](http://blast.ncbi.nlm.nih.gov/Blast.cgi#Query_130275) \| 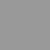 \| [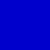](http://blast.ncbi.nlm.nih.gov/Blast.cgi#Query_130275) \| 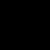 \| [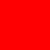](http://blast.ncbi.nlm.nih.gov/Blast.cgi#Query_130275) \| \| --- \| --- \| --- \| --- \| --- \| --- \| --- \|  \| 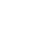 \| \| --- \| | | | | | |
| --- | --- | --- | --- | --- | --- | --- | --- | --- | --- | --- | --- | --- | --- | --- | --- | --- | --- | --- | --- | --- | --- | --- | --- | --- | --- | --- | --- | --- | --- | --- | --- | --- | --- | --- | --- | --- | --- | --- | --- | --- | --- | --- | --- | --- | --- | --- | --- | --- | --- | --- | --- | --- | --- | --- | --- |
|  |  |  |  |  |  |
| **Score** | **Expect** | **Method** | **Identities** | **Positives** | **Gaps** |
| 743 bits(1919) | 0.0 | Compositional matrix adjust. | 396/450(88%) | 408/450(90%) | 35/450(7%) |
|  |  |  |  |  |  |

Query 482 LTGTIEESNDSKPIDFEYHTAVEGAEGHAEGTIETEEDSIHVDFEESTHENSKHHADVVE 541

+TGT+EES+DSKPIDFEYHTAVEGAEGHAEGTIETEEDSIHVDFEESTHENSKHHADVVE

Sbjct 1 MTGTVEESDDSKPIDFEYHTAVEGAEGHAEGTIETEEDSIHVDFEESTHENSKHHADVVE 60

Query 542 YEEDTNPGGGQVTTESNLVEFDEDSTKGIVTGAVSDHTTIEDTKEYTTESNLIELVDELP 601

YEEDTNPGGGQVTTESNLVEFDE+STKGIVTGAVSDHTT+EDTKEYTTESNLIELVDELP

Sbjct 61 YEEDTNPGGGQVTTESNLVEFDEESTKGIVTGAVSDHTTVEDTKEYTTESNLIELVDELP 120

Query 602 EEHGQAQGPIEEITENNHHISHSGLGTENGHGNYGVIEEIEENSHVDIKSELGYEGGQNS 661

EEHGQAQGPIEEITENNHHISHSGLGTENGHGNYGVI+EIEENSHVDIKSELGYEGGQNS

Sbjct 121 EEHGQAQGPIEEITENNHHISHSGLGTENGHGNYGVIDEIEENSHVDIKSELGYEGGQNS 180

Query 662 GNQSFEEDTEEDKPKYEQGGNIVDIDFDSVPQIHGQNNGNQSFEEDTEKDKPKYEQGGNI 721

GNQSFEEDTEEDKPKYEQGGNIVDIDFDSVPQIHGQNNGNQSFEEDTE+DKPKYEQGGNI

Sbjct 181 GNQSFEEDTEEDKPKYEQGGNIVDIDFDSVPQIHGQNNGNQSFEEDTEEDKPKYEQGGNI 240

Query 722 IDIDFDSVPHIHGFNKHTEIIEEDTNKDKPNYQFGGHNSVDFEEDTLPQVSGHNEGQQTI 781

IDIDFDSVP IHGFNKH EIIEEDTNKDKPNYQFGGHNSVDFEEDTLP+VSG NEGQQTI

Sbjct 241 IDIDFDSVPQIHGFNKHNEIIEEDTNKDKPNYQFGGHNSVDFEEDTLPKVSGQNEGQQTI 300

Query 782 EEDTTPPIVPPTPPTPEV----------------------------PSEPETPTPPTPEV 813

EEDT TPPTPEV PSEPETP PPTPEV

Sbjct 301 EEDT-------TPPTPEVPSEPETPTPPTPEVPSEPGEPTPPTPEVPSEPETPVPPTPEV 353

Query 814 PTEPGKPIPPAKEEPKKPSKPVEQGKVVTPVIEINEKVKAVVPTKKAQSKKSELPETGGE 873

P+EPGKP+PPAKEEPKKPSKPVEQGKVVTPVIEINEKVKAV PTK+ QSKKSELPETGGE

Sbjct 354 PSEPGKPVPPAKEEPKKPSKPVEQGKVVTPVIEINEKVKAVAPTKQKQSKKSELPETGGE 413

Query 874 ESTNNGMLFGGLFSILGLALLRRNKKNHKA 903

ESTN G+LFGGLFSILGLALLRRNKKNHKA

Sbjct 414 ESTNKGILFGGLFSILGLALLRRNKKNHKA 443

| **Score** | **Expect** | **Method** | **Identities** | **Positives** | **Gaps** |
| --- | --- | --- | --- | --- | --- |
| 48.1 bits(113) | 6e-10 | Compositional matrix adjust. | 74/245(30%) | 106/245(43%) | 58/245(23%) |

Query 469 LEFKSEPPVEKHELTGTIEESNDSKPIDFEYHTAVEGAEGHAE-GTIETEEDSIHVDFEE 527

+E E P E + G IEE ++ H+ + GH G I+ E++ HVD +

Sbjct 113 IELVDELPEEHGQAQGPIEEITENN--HHISHSGLGTENGHGNYGVIDEIEENSHVDIKS 170

Query 528 STHENSKHHADVVEYEEDTNPGGGQVTTESNLVEFDEDSTKGIVTGAVSDHTTIEDTKEY 587

+ YE GGQ S F+ED T ED +Y

Sbjct 171 E-----------LGYE------GGQ---NSGNQSFEED--------------TEEDKPKY 196

Query 588 TTESNLIEL-VDELPEEHGQAQG--PIEEITENNHHISHSGLGTENGHGNYGVIEEIEEN 644

N++++ D +P+ HGQ G EE TE + G G I +I+ +

Sbjct 197 EQGGNIVDIDFDSVPQIHGQNNGNQSFEEDTEEDKPKYEQG----------GNIIDIDFD 246

Query 645 SHVDIKSELGYEGGQNSGNQSFEEDTEEDKPKYEQGG-NIVDIDFDSVPQIHGQNNGNQS 703

S I G N N+ EEDT +DKP Y+ GG N VD + D++P++ GQN G Q+

Sbjct 247 SVPQIH-------GFNKHNEIIEEDTNKDKPNYQFGGHNSVDFEEDTLPKVSGQNEGQQT 299

Query 704 FEEDT 708

EEDT

Sbjct 300 IEEDT 304

| **Score** | **Expect** | **Method** | **Identities** | **Positives** | **Gaps** |
| --- | --- | --- | --- | --- | --- |
| 16.5 bits(31) | 3.7 | Compositional matrix adjust. | 12/41(29%) | 21/41(51%) | 1/41(2%) |

Query 109 SNVSEIKPRMKRSTDVTAVAEKEVVEETKATGTDVTNKVEV 149

SN+ E + AV++ VE+TK T+ +N +E+

Sbjct 76 SNLVEFDEESTKGIVTGAVSDHTTVEDTKEYTTE-SNLIEL 115

**Additional file 10c**. **Fibronectin-Binding Protein B FnBpB. Integrative** **Genomics Viewer (IGV ).**


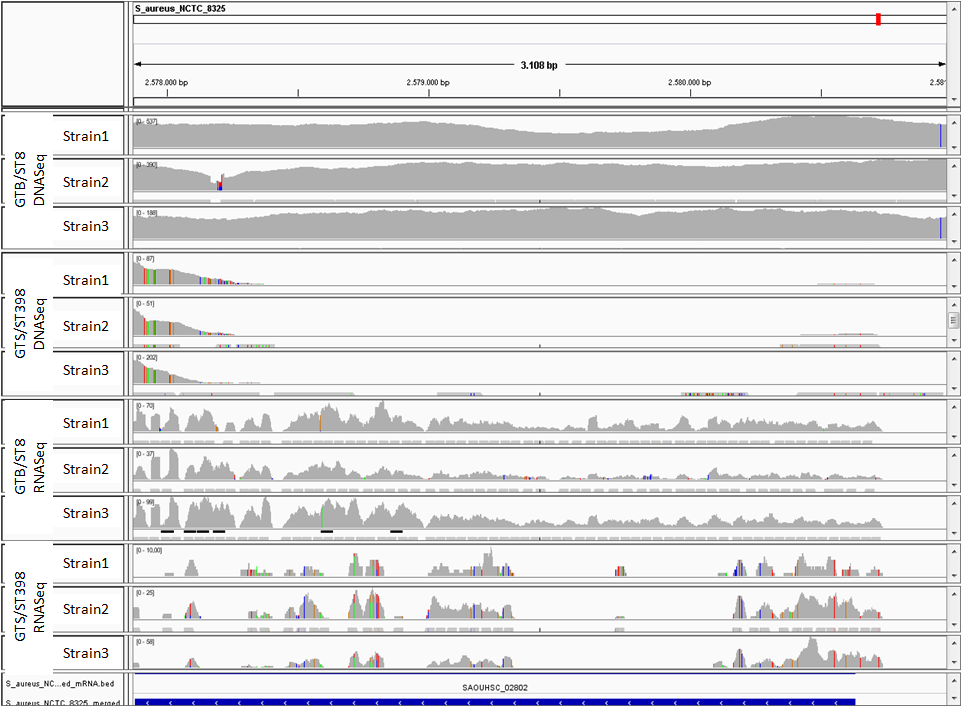

Supplement: Additional file 10: — Fibronectin-Binding Protein B FnBpB comparison between Staph. aureus GTB/ST8ra and GTS/ST398ra. a) FnBpB protein sequence for GTB/ST8ra (>fig|6666666.84847.peg.316 and GTS/ST398ra (>fig|6666666.84857.peg.2046, >fig|6666666.84857.peg.2047) b) Protein blast between GTB/ST8ra and GTS/ST398ra. c) Integrative Genomics Viewer (IGV) view comparison between three Staph. GTB/ST8 and three GTS/ST398 strains mapped on Staph. aureus NCTC 8325 strain with reads from DNASeq or RNA-Seq experiments. (DOCX 176 kb) [file 12866_2017_931_MOESM10_ESM.docx]
