# Supplementary material for: Genomic and transcriptomic comparison between Staphylococcus aureus strains associated with high and low within herd prevalence of intra-mammary infection
Source: BMC Microbiol. 2017 Jan 19;17:21. doi: 10.1186/s12866-017-0931-8 (PMC5247818; doi:10.1186/s12866-017-0931-8)

**Additional file 11.** Sequence alignment for a 1285 bp portion of fnbB gene (from position 1297up to 2582 of fnbB genes (NCBI accession number: CP000253, region: 2577879..2580632) in all six strains. In figure, gene sequence for GTS/ST398 (1-2-3), GTB/ST8 (1-2-3), and the NCTC8325 strain in the homologous position are presented.


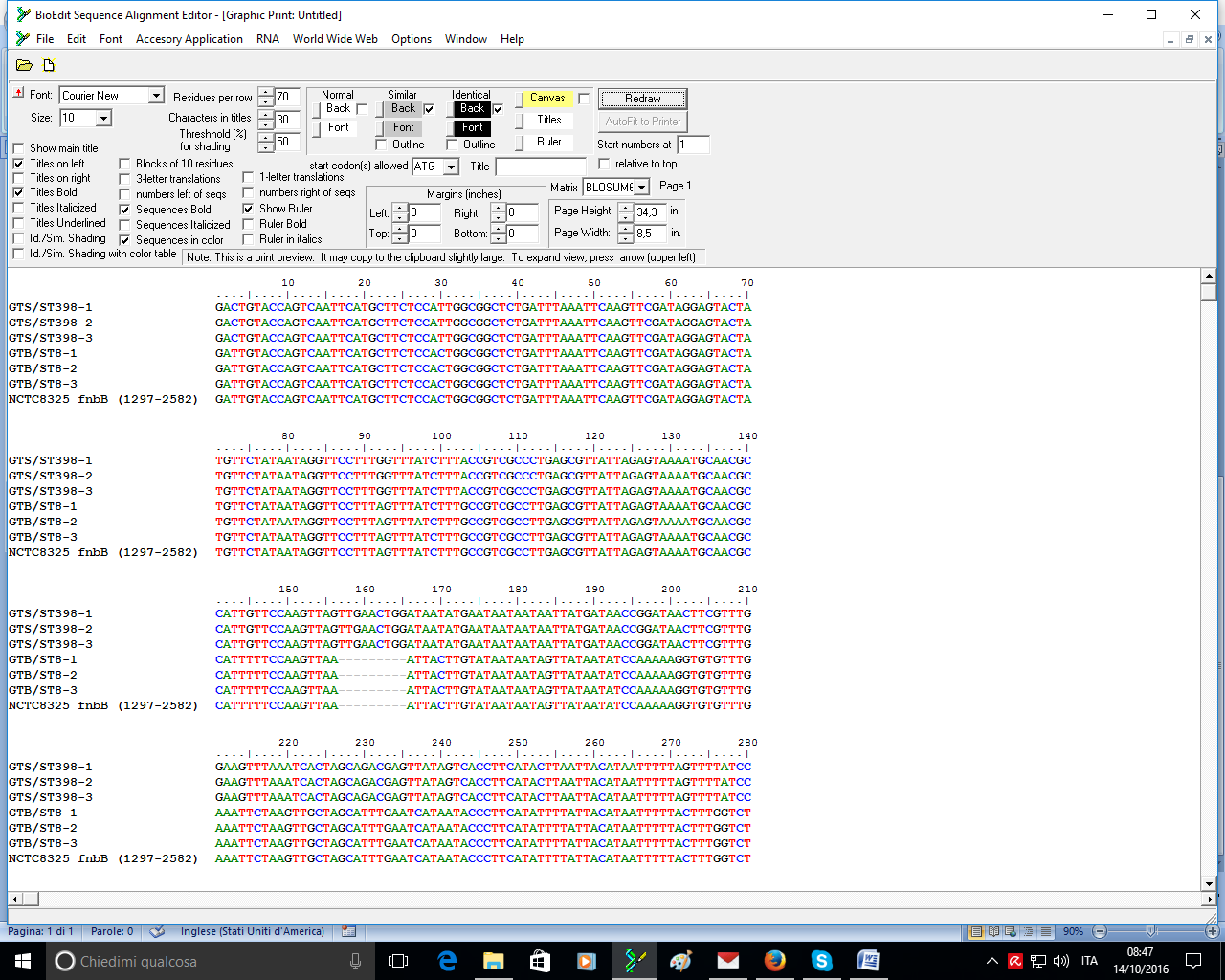


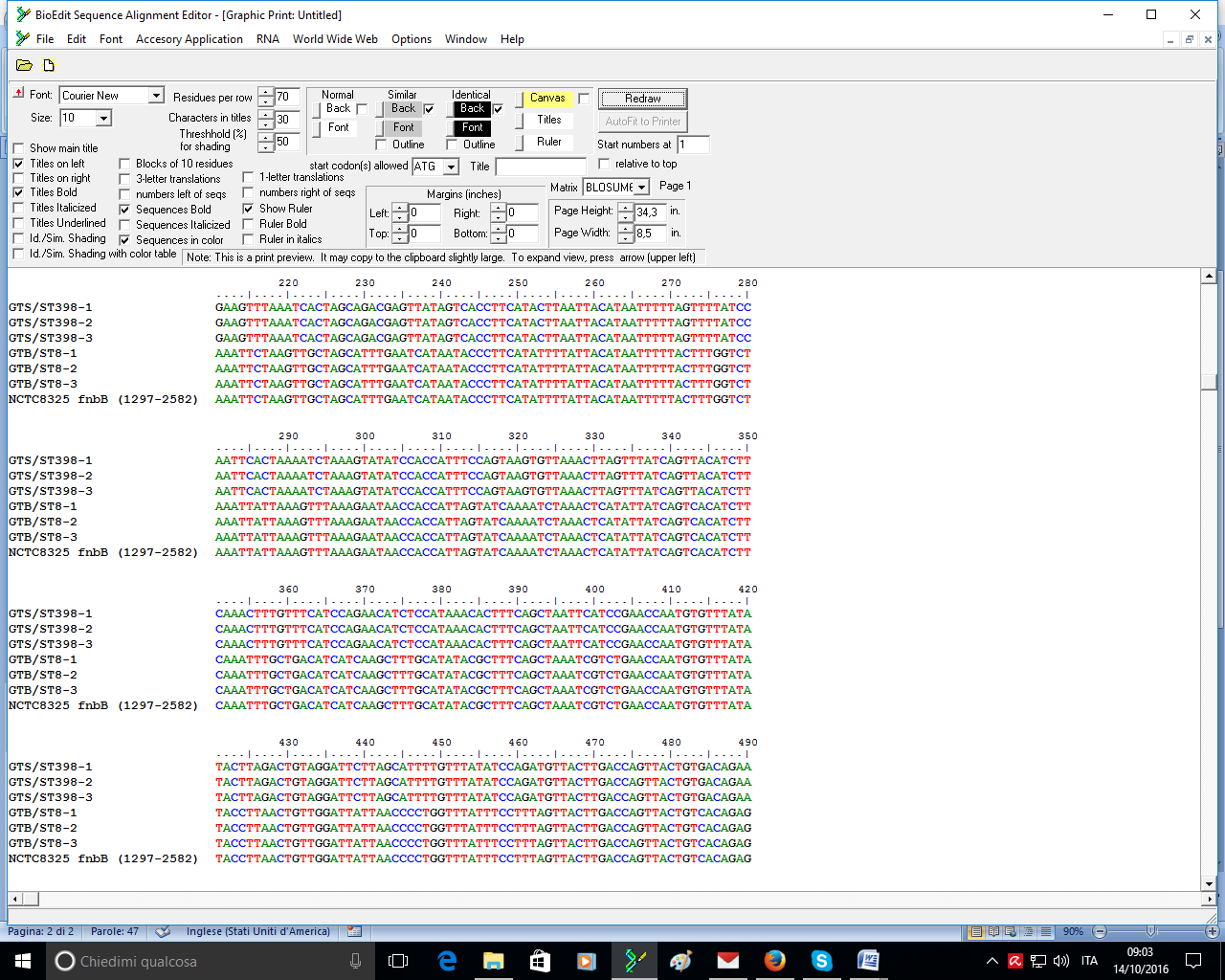


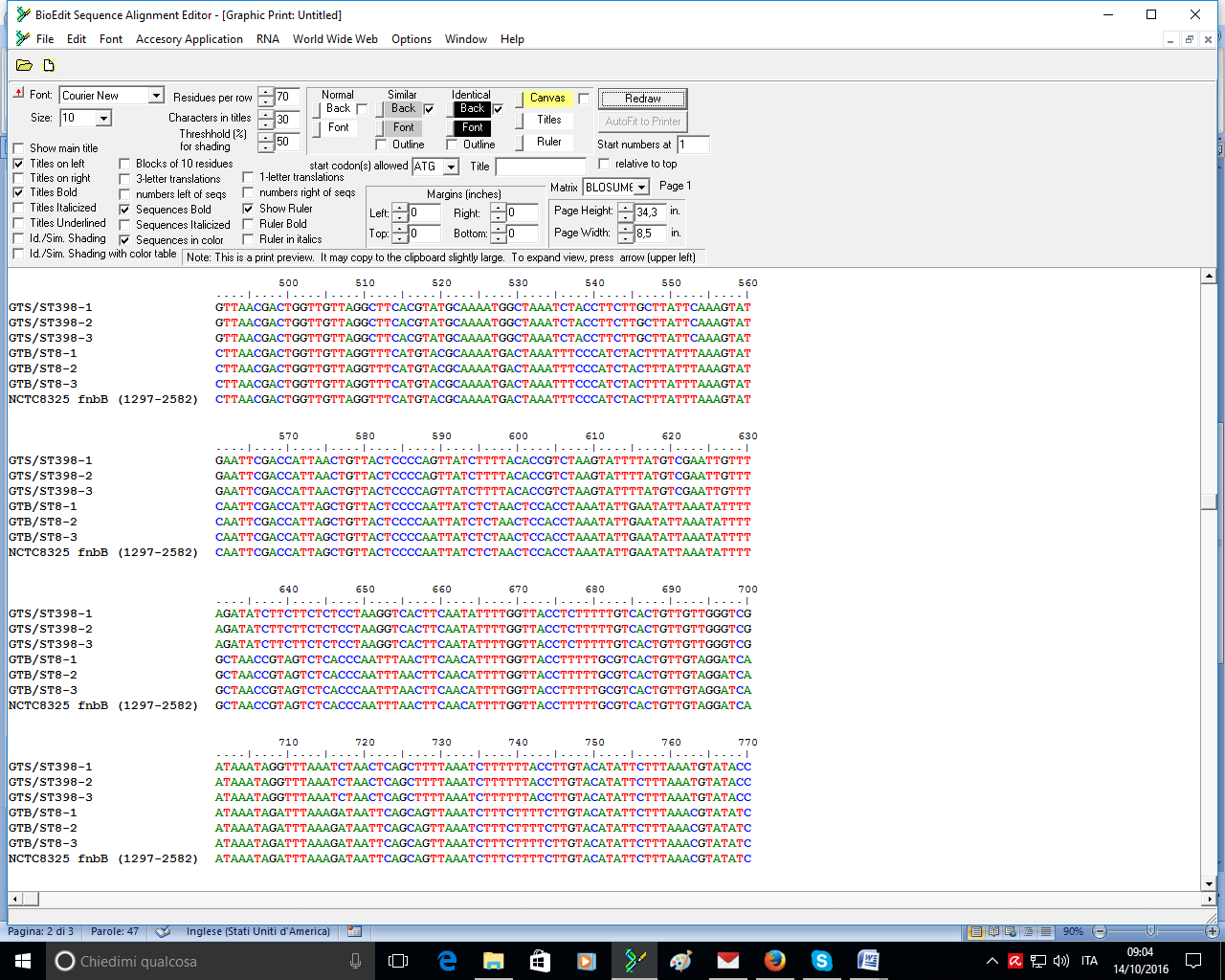


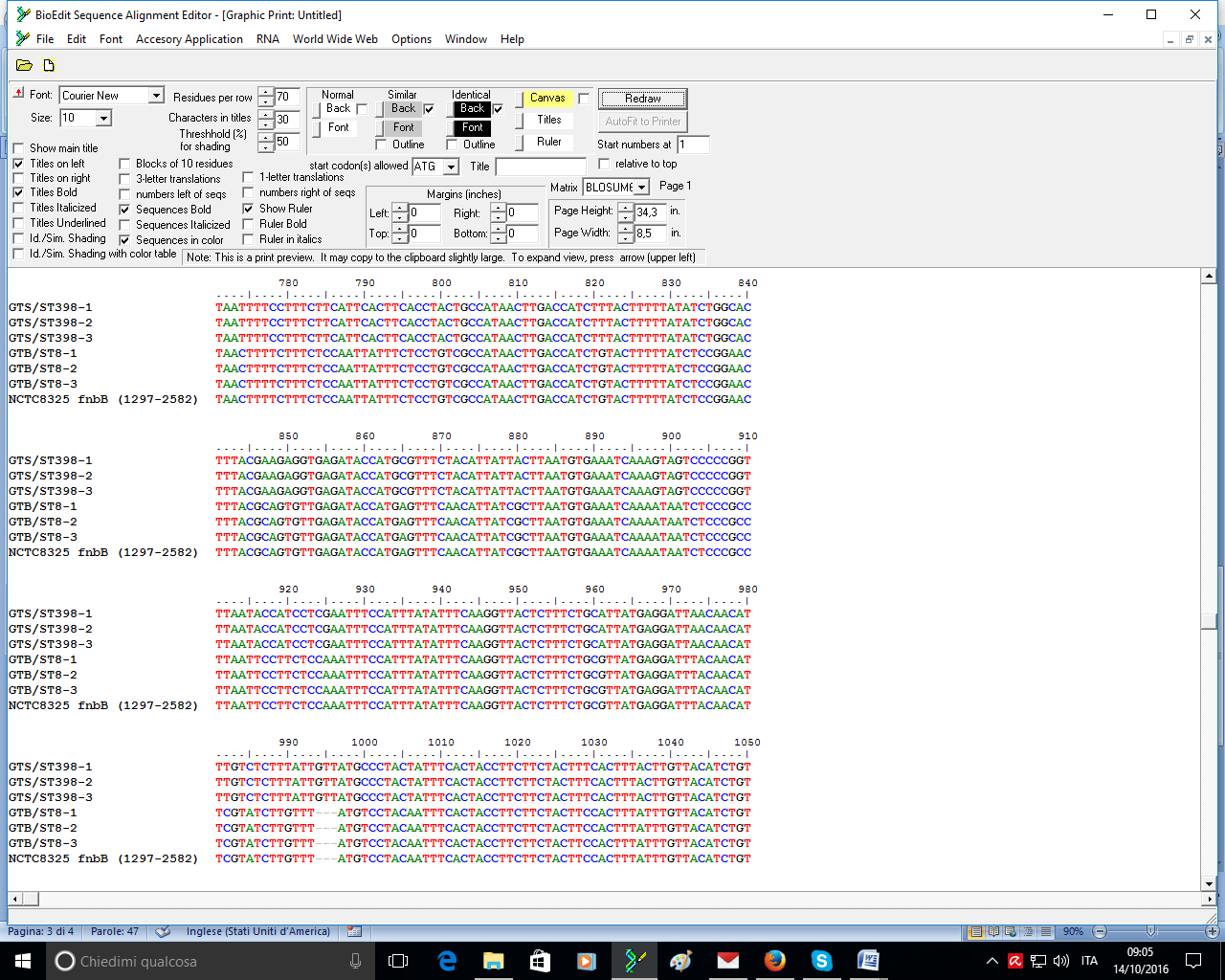


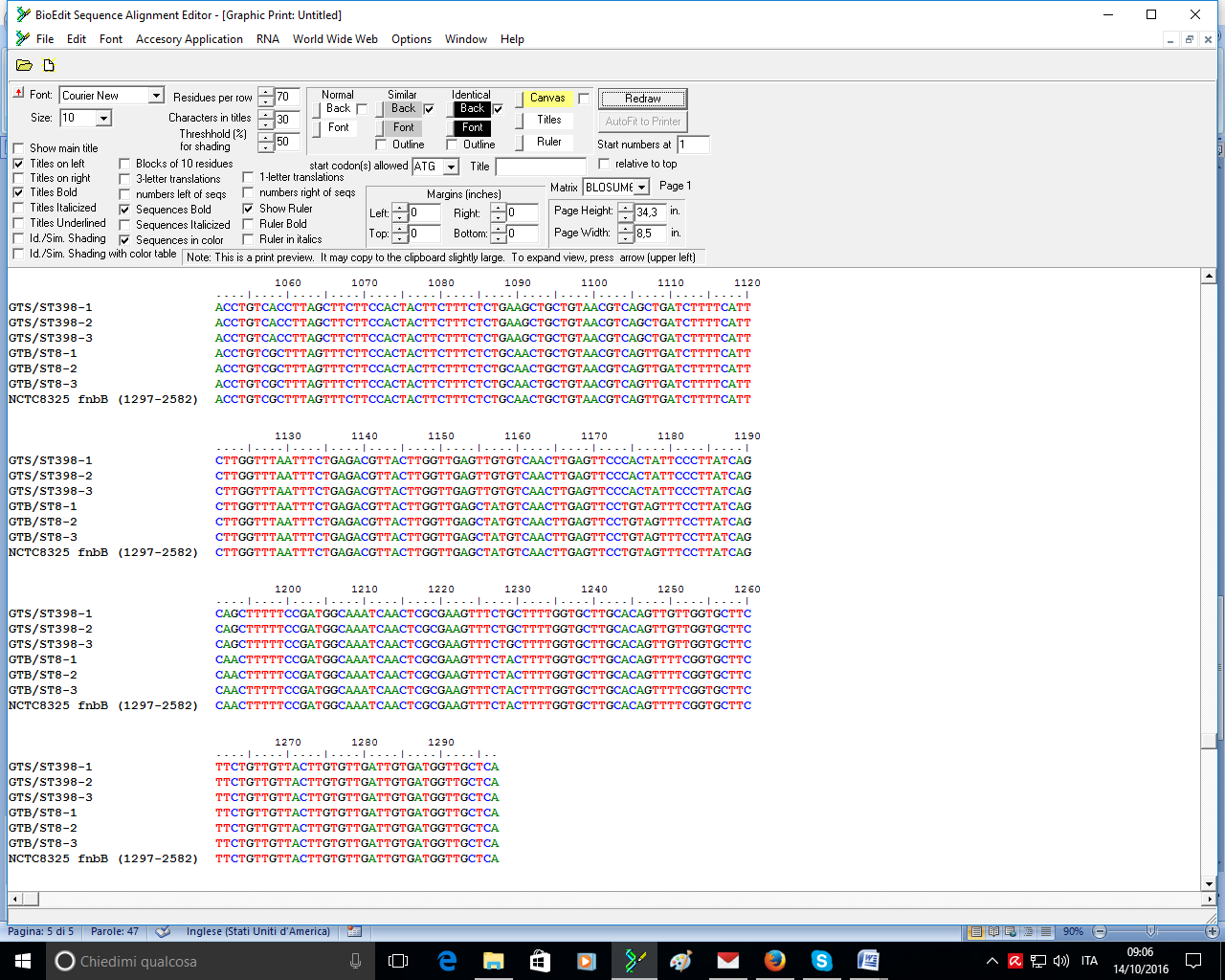

Supplement: Additional file 11: — Sequence alignment for a 1285 bp portion of fnbB gene (from position 1297up to 2582; NCBI accession number: CP000253, region: 2577879.....2580632) in all the six strains. In figure, gene sequence for GTS/ST398 (1-2-3), GTB/ST8 (1-2-3), and the NCTC8325 strain in the homologous position are presented. (DOCX 670 kb) [file 12866_2017_931_MOESM11_ESM.docx]
