# Supplementary material for: Genomic and transcriptomic comparison between Staphylococcus aureus strains associated with high and low within herd prevalence of intra-mammary infection
Source: BMC Microbiol. 2017 Jan 19;17:21. doi: 10.1186/s12866-017-0931-8 (PMC5247818; doi:10.1186/s12866-017-0931-8)

**Additional file 12a**. **clumping factor B ClfB. Integrative** **Genomics Viewer (IGV ).**

**
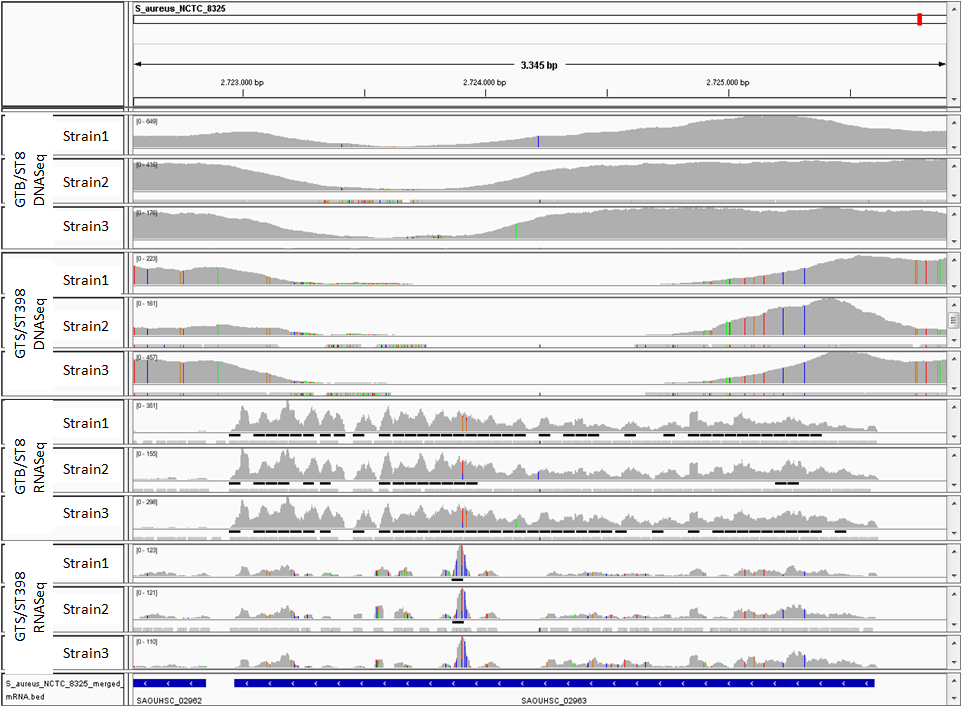
**

**Additional file 12b.** P**rotein A IsdA. Integrative** **Genomics Viewer (IGV ).**


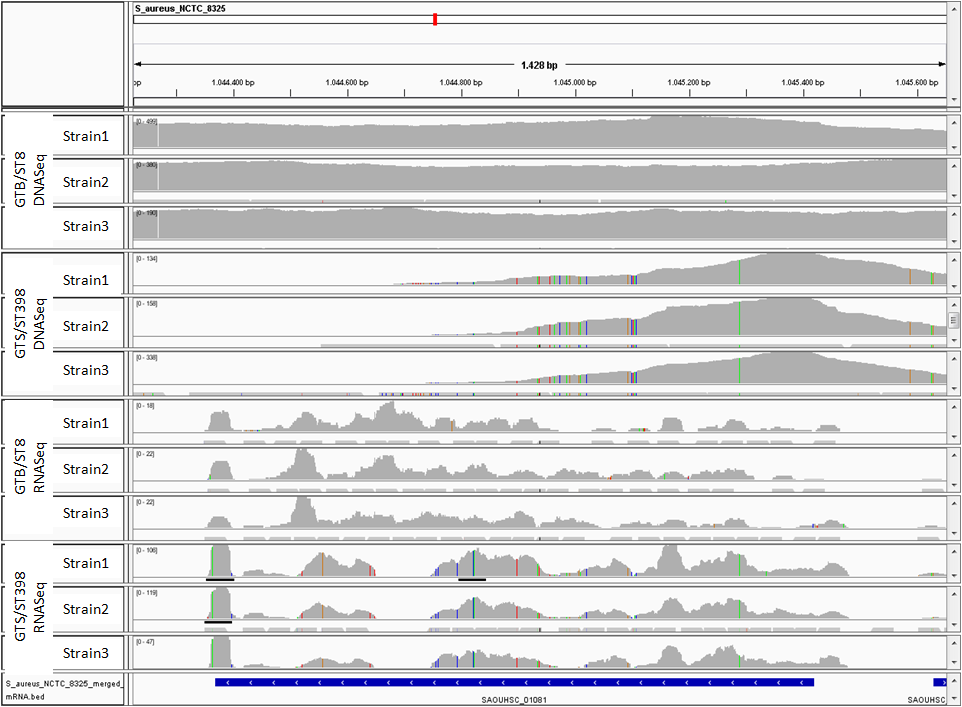

Supplement: Additional file 12: — Integrative Genomics Viewer (IGV) view comparison of a) clumping factor B ClfB and b) protein A IsdA, between Staph. aureus three GTB/ST8 and three GTS/ST398 strains mapped on Staph. aureus NCTC 8325 strain with reads from DNASeq or RNASeq experiment. (DOCX 316 kb) [file 12866_2017_931_MOESM12_ESM.docx]
