# Supplementary material for: Genomic and transcriptomic comparison between Staphylococcus aureus strains associated with high and low within herd prevalence of intra-mammary infection
Source: BMC Microbiol. 2017 Jan 19;17:21. doi: 10.1186/s12866-017-0931-8 (PMC5247818; doi:10.1186/s12866-017-0931-8)
Supplement: Additional file 13: — Serine-aspartate repeat-containing protein C/D/E SdrC/D/E and eap, map protein EaP/MaP comparison between Staph. aureus GTB/ST8ra and GTS/ST398ra. a) SdrC/D/E protein sequenze for GTB/ST8ra (>fig|6666666.84847.peg.2863) and GTS/ST398ra (>fig|6666666.84857.peg.510); Eap/Map protein sequence for GTB/ST8ra (>fig|6666666.84847.peg.1290) and GTS/ST398ra (>fig|6666666.84857.peg.609). b) Protein blast for SdrC/D/E and Eap/Map between GTB/ST8ra and GTS/ST398ra. (DOCX 42 kb) [file 12866_2017_931_MOESM13_ESM.docx]

**Additional file 13a**.

**Genotype GTB/ST8ra**

SdrC/D/E

>fig|6666666.84847.peg.2863

MNNKKTATNRKGMIPNRLNKFSIRKYSVGTASILVGTTLIFGLSGHEAKAAEHTNGELNQSKNETTAPSENKTTKKVDSRQLKDNTQTATADQPKVTMSDSATVKETSSNMQSPQNATANQSTTKTSNVTTNDKSSTTYSNETDKSNLTQAKDVSTTPKTTTIKPRTLNRMAVNTVAAPQQGTNVNDKVHFSNIDIAIDKGHVNQTTGKTEFWATSSDVLKLKANYTIDDSVKEGDTFTFKYGQYFRPGSVRLPSQTQNLYNAQGNIIAKGIYDSTTNTTTYTFTNYVDQYTNVRGSFEQVAFAKRKNATTDKTAYKMEVTLGNDTYSEEIIVDYGNKKAQPLISSTNYINNEDLSRNMTAYVNQPKNTYTKQTFVTNLTGYKFNPNAKNFKIYEVTDQNQFVDSFTPDTSKLKDVTDQFDVIYSNDNKTATVDLMKGQTSSNKQYIIQQVAYPDNSSTDNGKIDYTLDTDKTKYSWSNSYSNVNGSSTANGDQKKYNLGDYVWEDTNKDGKQDANEKGIKGVYVILKDSNGKELDRTTTDENGKYQFTGLSNGTYSVEFSTPAGYTPTTANVGTDDAVDSDGLTTTGVIKDADNMTLDSGFYKTPKYSLGDYVWYDSNKDGKQDSTEKGIKGVKVTLQNEKGEVIGTTETDENGKYRFDNLDSGKYKVIFEKPAGLTQTGTNTTEDDKDADGGEVDVTITDHDDFTLDNGYYEEETSDSDSDSDSDSDSDSDSDSDSDSDSDSDSDSDSDSDSDSDSDSDSDSDSDSDSDSDSDSDSDSDSDSDSDSDSDSDSDSDSDSDSDNDSDSDSDSDSDAGKHTPAKPMSTVKDQHKTAKALPETGSENNNSNNGTLFGGLFAALGSLLLFGRRKKQNK

Eap/Map

>fig|6666666.84847.peg.1290

MKFKSLITTTLALGVLASTGANFNNNEASAAAKPLDKSSSSLHHGYSKVHVPYAITVNGTSQNILSSLTFNKNQNISYKDLEDRVKSVLKSDRGISDIDLRLSKQAKYTVYFKNGTKKVIDLKAGIYTADLINTSEIKAININVDTKKQVEDKKKDKANYQVPYTITVNGTSQNILSNLTFNKNQNISYKDLEDKVKSVLESNRGITDVDLRLSKQAKYTVNFKNGTKKVIDLKSGIYTANLINSSDIKSININVDTKKHIENKAKRNYQVPYSINLNGTSTNILSNLSFSNKPWTNYKNLTSQIKSVLKHDRGISEQDLKYAKKAYYTVYFKNGGKRILQLNSKNYTANLVHAKDVKRIEITVKTGTKAKADRYVPYTIAVNGTSTPILSDLKFTGDPRVGYKDISKKVKSVLKHDRGIGERELKYAKKATYTVHFKNGTKKVININSNISQLNLLYVQDIKKIDIDVKTGTKAKADSYVPYTIAVNGTSTPILSKLKISNKQLISYKYLNDKVKSVLKSERGISDLDLKFAKQAKYTVYFKNGKKQVVNLKSDIFTPNLFSAKDIKKIDIDVKQYTKSKKNK

**Genotype GTS/ST398ra**

SdrC/D/E

>fig|6666666.84857.peg.510

MINRDNKKAITKKGMISNRLNKFSIRKYTVGTASILVGTTLIFGLGNQEAKAAENTSTENAKQDEASASDNEEVVSETENNSTTENTSTNPIKKETNTDSQPETKEESTTSSTQKQQNNVTATTETKPQNIEKENVKPSTDKTATEDTSVILEEKKAPNNTNNDVTTKPSTSEIQTKPTTPQESTNIENSQPQPTPSKVDNQVTDATNPKEPVNVSKEELKNNPEKLKELVRNDSNTDRSTKPVATAPTSVAPKRLNAKMRFAVAQPAAVASNNVNDLIKVTKQTIKVGDGKDDVVAAHDGEEIEYDSEFTIDNKVKAGDTMTINYDKNVIPSDLTDKNDPIDITDPSGEVIAKGTFDKATKQITYTFTDYVDKYEDIKSRLTLYSYIDKKTVPNETSLNLTFATAGKETSQNVTVDYQDPMVHGDSNIQSIFTKLDEDKQNIEQQIYVNPLKKTATNTKVDIAGSQVDDYGNIKLGNGSTIIDQNTEIKVYKVNSNQQLPQSNRIYDFSQYEDVTSQFDNKKSFSNNVATLDFGNIDSAYIIKVVSKYTPTSDDELDIAQGASMRTTDKNNYYNYAGYSNFIVTSTDTGGGDGTVKPEEKLYKIGDYVWEDVDKDGVQGTDSKEKPMANVLVTLTYPDGTTKSVRTDANGHYEFGGLKDGETYTVKFETPAGYLPTKENGTTDGEKDSNGSSVTVKINGKDDMSLDTGFYKEPKYNLGDYVWEDTNKDGIQDANEPGIKDVKVTLKDSTGKIIGTTTTDASGKYKFTDLDNGNYTVEFETPAGYTPTLKNTTAEDKDSNGLTTTGVIKDADNMTLDSGFYKTPKYSLGDYVWYDSNKDGKQDSTEKGIKDVTVTLQNEKGEVIGTTKTDENGKYRFDNLDSGKYKVIFEKPAGLTQTGTNTTEDDKDADGGEVDVTITDHDDFTIDNGYFEEDTSDSDSDSDSDSDSDSDSDSDSDSDSDSDSDSDSDSDSDSDSDSDSDSDSDSDSDSDSDSDSDSDSDSDSDSDSDSDSDSDSDSDSDSDSDSDSDSDSDSDSDSDSDSDSDSDSDSDSDSDSDSDSDSDSDSDSDSDSDSDSDSDSDSDSDSDSDSDSDSDSDTGKHIPVKPMSATKDHDNKAKALPETGSENNGSNNATLFGGLFAALGSLLLFGRRKKQNK

Eap/Map

>fig|6666666.84857.peg.609

MTGTVEESDDSKPIDFEYHTAVEGAEGHAEGTIETEEDSIHVDFEESTHENSKHHADVVEYEEDTNPGGGQVTTESNLVEFDEESTKGIVTGAVSDHTTVEDTKEYTTESNLIELVDELPEEHGQAQGPIEEITENNHHISHSGLGTENGHGNYGVIDEIEENSHVDIKSELGYEGGQNSGNQSFEEDTEEDKPKYEQGGNIVDIDFDSVPQIHGQNNGNQSFEEDTEEDKPKYEQGGNIIDIDFDSVPQIHGFNKHNEIIEEDTNKDKPNYQFGGHNSVDFEEDTLPKVSGQNEGQQTIEEDTTPPTPEVPSEPETPTPPTPEVPSEPGEPTPPTPEVPSEPETPVPPTPEVPSEPGKPVPPAKEEPKKPSKPVEQGKVVTPVIEINEKVKAVAPTKQKQSKKSELPETGGEESTNKGILFGGLFSILGLALLRRNKKNHKA

**Additional file 13b**.

**Blast SdrC/D/E (Genotype GTB/ST8ra vs GTS/ST398ra)**

| \| 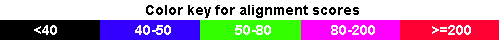 \| \| --- \|  \| 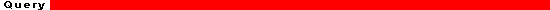 \| \| --- \|  \| 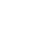 \| 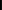 \| 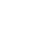 \| 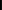 \| 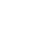 \| 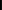 \| 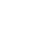 \| 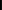 \| 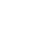 \| 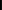 \| 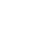 \| 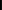 \| \| --- \| --- \| --- \| --- \| --- \| --- \| --- \| --- \| --- \| --- \| --- \| --- \|  \| 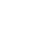 \| 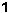 \| 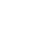 \| 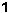 \| 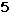 \| 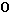 \| 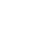 \| 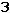 \| 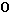 \| 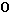 \| 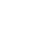 \| 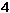 \| 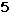 \| 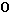 \| 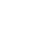 \| 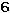 \| 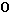 \| 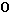 \| 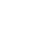 \| 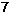 \| 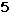 \| 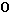 \| \| --- \| --- \| --- \| --- \| --- \| --- \| --- \| --- \| --- \| --- \| --- \| --- \| --- \| --- \| --- \| --- \| --- \| --- \| --- \| --- \| --- \| --- \|  \| 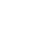 \| 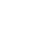 \| [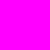](http://blast.ncbi.nlm.nih.gov/Blast.cgi#Query_75145) \| 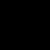 \| [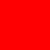](http://blast.ncbi.nlm.nih.gov/Blast.cgi#Query_75145) \| 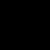 \| [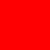](http://blast.ncbi.nlm.nih.gov/Blast.cgi#Query_75145) \| 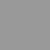 \| [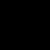](http://blast.ncbi.nlm.nih.gov/Blast.cgi#Query_75145) \| 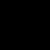 \| [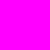](http://blast.ncbi.nlm.nih.gov/Blast.cgi#Query_75145) \| \| --- \| --- \| --- \| --- \| --- \| --- \| --- \| --- \| --- \| --- \| --- \|  \| 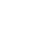 \| \| --- \| |
| --- | --- | --- | --- | --- | --- | --- | --- | --- | --- | --- | --- | --- | --- | --- | --- | --- | --- | --- | --- | --- | --- | --- | --- | --- | --- | --- | --- | --- | --- | --- | --- | --- | --- | --- | --- | --- | --- | --- | --- | --- | --- | --- | --- | --- | --- | --- | --- | --- |

Range 1: 713 to 935[Graphics](http://www.ncbi.nlm.nih.gov/projects/sviewer/?RID=XV4PR8R1114&id=lcl%7CQuery_75145&tracks=%5bkey:sequence_track,name:Sequence,display_name:Sequence,id:STD1,category:Sequence,annots:Sequence,ShowLabel:true%5d%5bkey:gene_model_track,CDSProductFeats:false%5d%5bkey:alignment_track,name:other%20alignments,annots:NG%20Alignments%7CRefseq%20Alignments%7CGnomon%20Alignments%7CUnnamed,shown:false%5d&v=702:946&appname=ncbiblast&link_loc=fromHSP) Next Match Previous Match

| Alignment statistics for **match #1** | | | | | |
| --- | --- | --- | --- | --- | --- |
| **Score** | **Expect** | **Method** | **Identities** | **Positives** | **Gaps** |
| 370 bits(951) | 3e-115 | Compositional matrix adjust. | 189/224(84%) | 200/224(89%) | 1/224(0%) |

Query 494 QKKYNLGDYVWEDTNKDGKQDANEKGIKGVYVILKDSNGKELDRTTTDENGKYQFTGLSN 553

+ KYNLGDYVWEDTNKDG QDANE GIK V V LKDS GK + TTTD +GKY+FT L N

Sbjct 713 EPKYNLGDYVWEDTNKDGIQDANEPGIKDVKVTLKDSTGKIIGTTTTDASGKYKFTDLDN 772

Query 554 GTYSVEFSTPAGYTPTTANVGTDDAVDSDGLTTTGVIKDADNMTLDSGFYKTPKYSLGDY 613

G Y+VEF TPAGYTPT N +D DS+GLTTTGVIKDADNMTLDSGFYKTPKYSLGDY

Sbjct 773 GNYTVEFETPAGYTPTLKNTTAEDK-DSNGLTTTGVIKDADNMTLDSGFYKTPKYSLGDY 831

Query 614 VWYDSNKDGKQDSTEKGIKGVKVTLQNEKGEVIGTTETDENGKYRFDNLDSGKYKVIFEK 673

VWYDSNKDGKQDSTEKGIK V VTLQNEKGEVIGTT+TDENGKYRFDNLDSGKYKVIFEK

Sbjct 832 VWYDSNKDGKQDSTEKGIKDVTVTLQNEKGEVIGTTKTDENGKYRFDNLDSGKYKVIFEK 891

Query 674 PAGLTQTGTNTTEDDKDADGGEVDVTITDHDDFTLDNGYYEEET 717

PAGLTQTGTNTTEDDKDADGGEVDVTITDHDDFT+DNGY+EE+T

Sbjct 892 PAGLTQTGTNTTEDDKDADGGEVDVTITDHDDFTIDNGYFEEDT 935

Range 2: 153 to 822[Graphics](http://www.ncbi.nlm.nih.gov/projects/sviewer/?RID=XV4PR8R1114&id=lcl%7CQuery_75145&tracks=%5bkey:sequence_track,name:Sequence,display_name:Sequence,id:STD1,category:Sequence,annots:Sequence,ShowLabel:true%5d%5bkey:gene_model_track,CDSProductFeats:false%5d%5bkey:alignment_track,name:other%20alignments,annots:NG%20Alignments%7CRefseq%20Alignments%7CGnomon%20Alignments%7CUnnamed,shown:false%5d&v=120:855&appname=ncbiblast&link_loc=fromHSP) Next Match Previous Match [First Match](http://blast.ncbi.nlm.nih.gov/Blast.cgi#hspQuery_75145_1)

| Alignment statistics for **match #2** | | | | | |
| --- | --- | --- | --- | --- | --- |
| **Score** | **Expect** | **Method** | **Identities** | **Positives** | **Gaps** |
| 268 bits(684) | 2e-79 | Compositional matrix adjust. | 244/709(34%) | 350/709(49%) | 79/709(11%) |

Query 46 HEAKAAEHTNGELNQSKNETTAPSENKTTKKVDSRQLKDNTQTATADQPKVTMSDSATVK 105

E KA +TN ++ TT PS ++ K + Q N + + QP+ T S

Sbjct 153 EEKKAPNNTNNDV------TTKPSTSEIQTKPTTPQESTNIENS---QPQPTPSKVDNQV 203

Query 106 ETSSNMQSPQNATANQ---STTKTSNVTTNDKSSTTYSNETDKSNLTQAKDVSTTPKTTT 162

++N + P N + + + K + ND + TD+S K V+T P T+

Sbjct 204 TDATNPKEPVNVSKEELKNNPEKLKELVRNDSN-------TDRS----TKPVATAP--TS 250

Query 163 IKPRTLNRMAVNTVAAPQQ--GTNVNDKVHFSNIDIAIDKGHVNQTTGKTEFWATSSDVL 220

+ P+ LN VA P NVND + + I + G + A + +

Sbjct 251 VAPKRLNAKMRFAVAQPAAVASNNVNDLIKVTKQTIKVGDGKDDVVA------AHDGEEI 304

Query 221 KLKANYTIDDSVKEGDTFTFKYGQYFRPGSVRLPSQTQNLYNAQGNIIAKGIYDSTTNTT 280

+ + +TID+ VK GDT T Y + P + + ++ + G +IAKG +D T

Sbjct 305 EYDSEFTIDNKVKAGDTMTINYDKNVIPSDLTDKNDPIDITDPSGEVIAKGTFDKATKQI 364

Query 281 TYTFTNYVDQYTNVRGSFEQVAFAKRKNATTDKTAYKMEVTLGNDTYSEEIIVDY----- 335

TYTFT+YVD+Y +++ ++ +K + + T G +T S+ + VDY

Sbjct 365 TYTFTDYVDKYEDIKSRLTLYSYIDKKTVPNETSLNLTFATAGKET-SQNVTVDYQDPMV 423

Query 336 -GNKKAQPLISSTNYINNEDLSRNMTAYVNQPKNTYTKQTFVTNLTGYKFN--PNAK--- 389

G+ Q + + ++ + + YVN K T T ++ G + + N K

Sbjct 424 HGDSNIQSIFTK---LDEDKQNIEQQIYVNPLKKTATNTKV--DIAGSQVDDYGNIKLGN 478

Query 390 ---------NFKIYEVTDQNQFVDS-FTPDTSKLKDVTDQFDVIYSNDNKTATVDLMKGQ 439

K+Y+V Q S D S+ +DVT QFD S N AT+D G

Sbjct 479 GSTIIDQNTEIKVYKVNSNQQLPQSNRIYDFSQYEDVTSQFDNKKSFSNNVATLDF--GN 536

Query 440 TSSNKQYIIQQVAYPDNSSTDNGKIDY---TLDTDKTKYSWSNSYSNV--------NGSS 488

S YII+ V+ +S D I TDK Y YSN G

Sbjct 537 IDS--AYIIKVVSKYTPTSDDELDIAQGASMRTTDKNNYYNYAGYSNFIVTSTDTGGGDG 594

Query 489 TANGDQKKYNLGDYVWEDTNKDGKQ--DANEKGIKGVYVILKDSNGKELDRTTTDENGKY 546

T ++K Y +GDYVWED +KDG Q D+ EK + V V L +G TD NG Y

Sbjct 595 TVKPEEKLYKIGDYVWEDVDKDGVQGTDSKEKPMANVLVTLTYPDGT-TKSVRTDANGHY 653

Query 547 QFTGLSNG-TYSVEFSTPAGYTPTTANVGTDDAVDSDGLTTTGVIKDADNMTLDSGFYKT 605

+F GL +G TY+V+F TPAGY PT N TD DS+G + T I D+M+LD+GFYK

Sbjct 654 EFGGLKDGETYTVKFETPAGYLPTKENGTTDGEKDSNGSSVTVKINGKDDMSLDTGFYKE 713

Query 606 PKYSLGDYVWYDSNKDGKQDSTEKGIKGVKVTLQNEKGEVIGTTETDENGKYRFDNLDSG 665

PKY+LGDYVW D+NKDG QD+ E GIK VKVTL++ G++IGTT TD +GKY+F +LD+G

Sbjct 714 PKYNLGDYVWEDTNKDGIQDANEPGIKDVKVTLKDSTGKIIGTTTTDASGKYKFTDLDNG 773

Query 666 KYKVIFEKPAGLTQTGTNTTEDDKDADGGEVDVTITDHDDFTLDNGYYE 714

Y V FE PAG T T NTT +DKD++G I D D+ TLD+G+Y+

Sbjct 774 NYTVEFETPAGYTPTLKNTTAEDKDSNGLTTTGVIKDADNMTLDSGFYK 822

Range 3: 1099 to 1157[Graphics](http://www.ncbi.nlm.nih.gov/projects/sviewer/?RID=XV4PR8R1114&id=lcl%7CQuery_75145&tracks=%5bkey:sequence_track,name:Sequence,display_name:Sequence,id:STD1,category:Sequence,annots:Sequence,ShowLabel:true%5d%5bkey:gene_model_track,CDSProductFeats:false%5d%5bkey:alignment_track,name:other%20alignments,annots:NG%20Alignments%7CRefseq%20Alignments%7CGnomon%20Alignments%7CUnnamed,shown:false%5d&v=1097:1159&appname=ncbiblast&link_loc=fromHSP) Next Match Previous Match [First Match](http://blast.ncbi.nlm.nih.gov/Blast.cgi#hspQuery_75145_1)

| Alignment statistics for **match #3** | | | | | |
| --- | --- | --- | --- | --- | --- |
| **Score** | **Expect** | **Method** | **Identities** | **Positives** | **Gaps** |
| 108 bits(270) | 5e-28 | Compositional matrix adjust. | 49/59(83%) | 49/59(83%) | 0/59(0%) |

Query 817 GKHTPAKPMSTVKDQHKTAKALPETGSENNNSNNGTLFGGLFAALGSLLLFGRRKKQNK 875

GKH P KPMS KD AKALPETGSENN SNN TLFGGLFAALGSLLLFGRRKKQNK

Sbjct 1099 GKHIPVKPMSATKDHDNKAKALPETGSENNGSNNATLFGGLFAALGSLLLFGRRKKQNK 1157

Range 4: 7 to 187[Graphics](http://www.ncbi.nlm.nih.gov/projects/sviewer/?RID=XV4PR8R1114&id=lcl%7CQuery_75145&tracks=%5bkey:sequence_track,name:Sequence,display_name:Sequence,id:STD1,category:Sequence,annots:Sequence,ShowLabel:true%5d%5bkey:gene_model_track,CDSProductFeats:false%5d%5bkey:alignment_track,name:other%20alignments,annots:NG%20Alignments%7CRefseq%20Alignments%7CGnomon%20Alignments%7CUnnamed,shown:false%5d&v=0:196&appname=ncbiblast&link_loc=fromHSP) Next Match Previous Match [First Match](http://blast.ncbi.nlm.nih.gov/Blast.cgi#hspQuery_75145_1)

| Alignment statistics for **match #4** | | | | | |
| --- | --- | --- | --- | --- | --- |
| **Score** | **Expect** | **Method** | **Identities** | **Positives** | **Gaps** |
| 98.2 bits(243) | 1e-24 | Compositional matrix adjust. | 79/193(41%) | 104/193(53%) | 24/193(12%) |

Query 5 KTATNRKGMIPNRLNKFSIRKYSVGTASILVGTTLIFGLSGHEAKAAEHTNGELNQSKNE 64

K A +KGMI NRLNKFSIRKY+VGTASILVGTTLIFGL EAKAAE+T+ E N ++E

Sbjct 7 KKAITKKGMISNRLNKFSIRKYTVGTASILVGTTLIFGLGNQEAKAAENTSTE-NAKQDE 65

Query 65 TTAPSENKTTKKVDSRQLKDNTQTATADQPKVTMSDSATVKE-TSSNMQSPQNATANQST 123

+A + + ++ +NT T + T S T +E T+S+ Q QN +

Sbjct 66 ASASDNEEVVSETENNSTTENTSTNPIKKETNTDSQPETKEESTTSSTQKQQNNVTATTE 125

Query 124 TKTSNV-TTNDKSSTTYSNETDKSNLTQAK--------DVSTTPKTTTI--KPRTLNRMA 172

TK N+ N K ST + D S + + K DV+T P T+ I KP T

Sbjct 126 TKPQNIEKENVKPSTDKTATEDTSVILEEKKAPNNTNNDVTTKPSTSEIQTKPTT----- 180

Query 173 VNTVAAPQQGTNV 185

PQ+ TN+

Sbjct 181 ------PQESTNI 187

Range 5: 612 to 631[Graphics](http://www.ncbi.nlm.nih.gov/projects/sviewer/?RID=XV4PR8R1114&id=lcl%7CQuery_75145&tracks=%5bkey:sequence_track,name:Sequence,display_name:Sequence,id:STD1,category:Sequence,annots:Sequence,ShowLabel:true%5d%5bkey:gene_model_track,CDSProductFeats:false%5d%5bkey:alignment_track,name:other%20alignments,annots:NG%20Alignments%7CRefseq%20Alignments%7CGnomon%20Alignments%7CUnnamed,shown:false%5d&v=612:631&appname=ncbiblast&link_loc=fromHSP) Next Match Previous Match [First Match](http://blast.ncbi.nlm.nih.gov/Blast.cgi#hspQuery_75145_1)

| Alignment statistics for **match #5** | | | | | |
| --- | --- | --- | --- | --- | --- |
| **Score** | **Expect** | **Method** | **Identities** | **Positives** | **Gaps** |
| 17.3 bits(33) | 6.0 | Compositional matrix adjust. | 8/20(40%) | 10/20(50%) | 0/20(0%) |

Query 809 DSDSDSDAGKHTPAKPMSTV 828

D D D G + KPM+ V

Sbjct 612 DVDKDGVQGTDSKEKPMANV 631

**Blast Eap/Map (Genotype GTB/ST8ra vs GTS/ST398ra)**

| \| 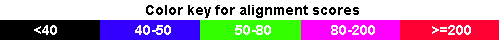 \| \| --- \|  \| 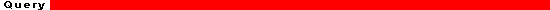 \| \| --- \|  \| 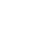 \| 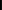 \| 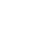 \| 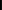 \| 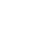 \| 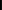 \| 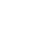 \| 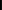 \| 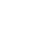 \| 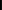 \| 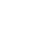 \| 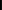 \| \| --- \| --- \| --- \| --- \| --- \| --- \| --- \| --- \| --- \| --- \| --- \| --- \|  \| 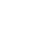 \| 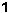 \| 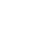 \| 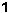 \| 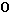 \| 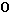 \| 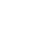 \| 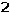 \| 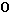 \| 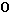 \| 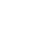 \| 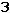 \| 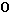 \| 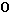 \| 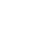 \| 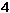 \| 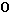 \| 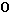 \| 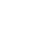 \| 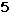 \| 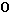 \| 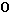 \| \| --- \| --- \| --- \| --- \| --- \| --- \| --- \| --- \| --- \| --- \| --- \| --- \| --- \| --- \| --- \| --- \| --- \| --- \| --- \| --- \| --- \| --- \|  \| 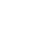 \| 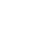 \| [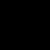](http://blast.ncbi.nlm.nih.gov/Blast.cgi#Query_17579) \| 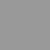 \| [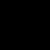](http://blast.ncbi.nlm.nih.gov/Blast.cgi#Query_17579) \| \| --- \| --- \| --- \| --- \| --- \|  \| 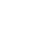 \| \| --- \| |
| --- | --- | --- | --- | --- | --- | --- | --- | --- | --- | --- | --- | --- | --- | --- | --- | --- | --- | --- | --- | --- | --- | --- | --- | --- | --- | --- | --- | --- | --- | --- | --- | --- | --- | --- | --- | --- | --- | --- | --- | --- | --- | --- |

Range 1: 122 to 273[Graphics](http://www.ncbi.nlm.nih.gov/projects/sviewer/?RID=XV4VAB46114&id=lcl%7CQuery_17579&tracks=%5bkey:sequence_track,name:Sequence,display_name:Sequence,id:STD1,category:Sequence,annots:Sequence,ShowLabel:true%5d%5bkey:gene_model_track,CDSProductFeats:false%5d%5bkey:alignment_track,name:other%20alignments,annots:NG%20Alignments%7CRefseq%20Alignments%7CGnomon%20Alignments%7CUnnamed,shown:false%5d&v=115:280&appname=ncbiblast&link_loc=fromHSP) Next Match Previous Match

| Alignment statistics for **match #1** | | | | | |
| --- | --- | --- | --- | --- | --- |
| **Score** | **Expect** | **Method** | **Identities** | **Positives** | **Gaps** |
| 20.8 bits(42) | 0.11 | Compositional matrix adjust. | 38/155(25%) | 61/155(39%) | 23/155(14%) |

Query 27 EASAAAKPLDKSSSSLHH------GYSKVHVPYAITVNGTSQN----ILSSLTFNKNQNI 76

E A P+++ + + HH G H Y + ++ +N I S L + QN

Sbjct 122 EHGQAQGPIEEITENNHHISHSGLGTENGHGNYGV-IDEIEENSHVDIKSELGYEGGQNS 180

Query 77 SYKDLE-DRVKSVLKSDRG--ISDIDL----RLSKQAKYTVYFKNGT---KKVIDLKAGI 126

+ E D + K ++G I DID ++ Q F+ T K + I

Sbjct 181 GNQSFEEDTEEDKPKYEQGGNIVDIDFDSVPQIHGQNNGNQSFEEDTEEDKPKYEQGGNI 240

Query 127 YTADLINTSEIKAININVDTKKQVEDKKKDKANYQ 161

D + +I N + + + ED KDK NYQ

Sbjct 241 IDIDFDSVPQIHGFNKHNEIIE--EDTNKDKPNYQ 273

Range 2: 218 to 245[Graphics](http://www.ncbi.nlm.nih.gov/projects/sviewer/?RID=XV4VAB46114&id=lcl%7CQuery_17579&tracks=%5bkey:sequence_track,name:Sequence,display_name:Sequence,id:STD1,category:Sequence,annots:Sequence,ShowLabel:true%5d%5bkey:gene_model_track,CDSProductFeats:false%5d%5bkey:alignment_track,name:other%20alignments,annots:NG%20Alignments%7CRefseq%20Alignments%7CGnomon%20Alignments%7CUnnamed,shown:false%5d&v=217:246&appname=ncbiblast&link_loc=fromHSP) Next Match Previous Match [First Match](http://blast.ncbi.nlm.nih.gov/Blast.cgi#hspQuery_17579_1)

| Alignment statistics for **match #2** | | | | | |
| --- | --- | --- | --- | --- | --- |
| **Score** | **Expect** | **Method** | **Identities** | **Positives** | **Gaps** |
| 16.9 bits(32) | 2.1 | Compositional matrix adjust. | 11/30(37%) | 13/30(43%) | 2/30(6%) |

Query 182 NKNQNISYKDLEDKVKSVLESNRGITDVDL 211

N NQ+ EDK K E I D+D

Sbjct 218 NGNQSFEEDTEEDKPK--YEQGGNIIDIDF 245
